# Diabetes care performance in Indonesia: a serial cross-sectional analysis of behavioral, clinical, and laboratory outcomes from 2013 to 2023

**DOI:** 10.1016/j.lanwpc.2025.101759

**Published:** 2025-11-23

**Authors:** Farizal Rizky Muharram, Julian Benedict Swannjo, Dicky Lavenus Tahapary, Sally Aman Nasution, Delvac Oceandy

**Affiliations:** a Global Health and Social Medicine, Harvard Medical School, Boston, USA; b Health System Center, ARC Institute, Jakarta, Indonesia; c Division of Endocrinology, Metabolism, and Diabetes, Department of Internal Medicine, Faculty of Medicine Universitas Indonesia, Dr. Cipto Mangunkusumo Hospital, Jakarta, Indonesia; d Metabolic, Cardiovascular, and Aging Research Cluster, The Indonesian Medical Education and Research Institute, Faculty of Medicine, Universitas Indonesia, Indonesia; e Department of Internal Medicine, Faculty of Medicine, Universitas Indonesia, Jakarta, Indonesia; f Division of Cardiovascular Sciences, Faculty of Biology, Medicine and Health, University of Manchester, United Kingdom

**Keywords:** Diabetes management, Non-communicable diseases, Indonesia

## Abstract

**Background:**

The growing diabetes burden in Indonesia necessitates a comprehensive understanding of national diabetes care performance, which remains inadequately characterized. Evaluating care quality across domains is essential to inform chronic disease policy and improve health outcomes. This study assesses trends in behavioral, clinical, and laboratory outcomes of diabetes care in Indonesia from 2013 to 2023.

**Methods:**

We conducted a serial cross-sectional analysis of pooled data from the 2013, 2018, and 2023 Indonesian national health surveys (N = 42,224 for behavioral-clinical and N = 2957 for laboratory outcomes). Diabetes care performance was assessed across behavioral (treatment, smoking, diet, activity), clinical (blood pressure, BMI, waist length), and laboratory (glucose, lipids, renal function) domains. Composite scores and multilevel models were used to identify geographic and sociodemographic disparities.

**Findings:**

Although linkage to diabetes care significantly improved from 68% to 92% between 2013 and 2023, performance in most other indicators remained stagnant or declined. In 2023, only 2.9% (95% CI 2.5–3.3%) met dietary fiber intake targets, 62.4% (95% CI 61.2–63.6%) achieved physical activity goals, and 83.9% (95% CI 82.9–84.8%) abstained from smoking. Clinical control was suboptimal, with 43.5% (95% CI 42.3–44.8%) meeting blood pressure targets and only 26.7% (95% CI 25.6–27.9%) and 33.1% (95% CI 32.0–34.3%) achieving BMI and waist circumference goals, respectively. Laboratory control was limited: only 25.2% (21.6–28.8%) achieved fasting glucose targets, 32.0% (95% CI 27.6–36.3%) had HbA1c <7%, and only 22.6% (95% CI 19.1–26.2%) met LDL-C goals. Fewer than 5% of participants met all behavioral-clinical or laboratory composite targets. Composite performance declined in nearly all provinces, with disparities linked to older age, male sex, lower education, and rural residence.

**Interpretation:**

Despite expanded healthcare coverage, Indonesia's diabetes care performance remains critically inadequate, particularly for achieving multiple targets. Strengthening national guidelines, embedding structured chronic care, and addressing social determinants are essential to improving diabetes outcomes.

**Funding:**

None.

**Research in context**

**Evidence before this study**

We conducted a literature review using PubMed and Google Scholar to identify studies published before July 15, 2025, on diabetes prevalence, diagnosis, and care in Indonesia. Search terms included ("Indonesia") AND ("diabetes" OR "diabetes management") AND ("prevalence" OR "HbA1c" OR "glycemic control"). Evidence shows that despite government efforts to implement diabetes management programs, only 66% of diagnosed individuals received both pharmacological and non-pharmacological treatments. Additionally, previous research showed poor diabetes care outcomes, with only 30% of patients achieving the recommended HbA1c target of <7%. However, limited studies have explored the multidimensional outcomes of diabetes care performance in the Indonesian population.

**Added value of this study**

• This is the first national study to evaluate trends in diabetes care performance in Indonesia over 10 years, using repeated, population-based data from the country's largest health surveys (Riskesdas 2013, 2018, and SKI 2023).
• It provides the most detailed assessment to date across behavioral, clinical, and laboratory domains, including composite indicators rarely used in national monitoring, offering a more holistic view of care quality.
• The study identifies critical equity gaps and system-level barriers by examining geographical variation and social determinants of care outcomes, informing more targeted chronic disease policy.
• The findings establish a national benchmark for diabetes management and offer practical insights to guide future reforms in Indonesia's primary care and chronic disease frameworks.

**Implications of all the available evidence**

Despite improved care linkage, major gaps persist in Indonesia's diabetes management, particularly in blood pressure, cholesterol control, and obesity-related outcomes. The rising prevalence of diabetes, combined with low achievement of comprehensive care targets (e.g., ABC and ABCN control), signals the need for urgent reforms. Policymakers and healthcare providers must prioritize integrated, outcome-based care strategies to improve long-term diabetes control and reduce health disparities nationwide.

## Introduction

The burden of diabetes in terms of prevalence, morbidity, and mortality poses a significant problem worldwide, including in Indonesia. An estimated 463 million adults aged 20–79 are affected by diabetes, and this number is expected to increase to 578 million people in 2030 worldwide.^1^ In Indonesia, where the demographic pyramid is gradually shifting towards a higher proportion of older adults, there is a notable rise in the prevalence of non-communicable diseases (NCDs), including diabetes.^2^ Based on the Indonesia Health Survey report, the prevalence of diabetes consistently increased from 5.7% in 2007 to 6.9% in 2013, then rose to 10.9% in 2018, and reached 11.7% in 2023, translated into 20 million. Geographic disparities existed in diabetes diagnosis rates in Indonesia across the subnational level; for example, 3.1% of Jakarta's population was diagnosed with diabetes, compared to only 0.2% in Papua Pegunungan. Moreover, data from the Indonesian Family Life Survey indicated a higher prevalence among people aged 50 years or older and a slightly higher prevalence among females than among males.^3^ This fact placed Indonesia in the top 10 countries with the highest increase in prevalence of diabetes.^1^

In response to this trend, diabetes has been designated as one of the four priority areas under the government's NCD agenda, as outlined in the National Medium-Term Development Plan (2020–2024).^4^ and the Strategic Plan of the Ministry of Health (2020–2024).^5^ Programs such as Posbindu (community-based integrated NCD posts) and Prolanis (chronic disease management program under the National Health Insurance scheme) have been implemented to strengthen diabetes education and management^5^; however, a significant gap in diabetes care exists,^6^ with only 66% of diagnosed individuals receiving both pharmacological and non-pharmacological treatment.^7^ The latest study on Indonesia's diabetes care performance in 2007 and 2014 reported unsatisfactory outcomes, with only 30% of diagnosed patients (±7,5% of the total diabetes population) achieving the HbA1c target of <7%.^7^^,^^8^

In addition, a more recent study reported that the majority of type 2 diabetes mellitus patients in Indonesia did not reach the HbA1c recommended target.^9^ This affects the cost of burden, where 74% of the total costs were used for the management of people with diabetes complications.^10^

The Indonesian Society of Endocrinology (PERKENI) suggests various glucose-lowering treatments as the first-line option depending on the patient's glycated hemoglobin (HbA1c) in combination with lifestyle modifications.^11^^,^^12^ Ensuring glycemic control, managing comorbidities including hypertension (blood pressure), body anthropometry (including body mass index and waist length), hyperlipidemia, and promoting smoking cessation are crucial in the successful diabetes treatment, significant targets.^13^ In addition, Indonesia's Ministry of Health has included diabetes management within its non-communicable diseases (NCD) program for the past decade. However, the impact of the program on Indonesia's diabetes care remains unclear due to the lack of comprehensive evidence. To address this problem, a comprehensive evaluation of diabetes care performance in Indonesia is clearly needed. This study, therefore, aims to assess trends in key aspects of diabetes management and treatment targets in Indonesia between 2013 and 2023.

## Methods

### Study design and population

This study is a serial cross-sectional analysis using Indonesian Health Research surveys conducted in 2013, 2018, and 2023 (Riskesdas in 2013 and 2018, and SKI in 2023). These surveys employ a complex, multistage stratified sampling design based on randomization in census blocks and households within. The sample was weighed to ensure representativeness at the national level. Details of the sampling framework and weighting were provided in Supplementary Text 1. Riskesdas and SKI comprise two data components: (1) interview and anthropometric data on all household samples and (2) laboratory data from a subsample of around 30,000 participants per year. The interview data are representative at the provincial and national levels, while the laboratory data are representative at the national level. Laboratory analyses included fasting plasma glucose (FPG), hemoglobin A1c (HbA1c, available only in the 2023 survey), and lipid profiles (LDL-C, HDL-C, and total cholesterol).

The population of this study is those who self-reported having diabetes from physician diagnosis (diagnosed diabetes). We conducted complete case analyses, excluding participants with missing information on exposures, outcomes, or covariates from the respective models (see Supplementary Figure 1). Across the three surveys, a total of 2,845,754 individuals completed the behavioral and clinical components, with 42,224 participants aged 15 years or older who reported a physician diagnosis of diabetes and were included in the behavioral and clinical analysis. For the laboratory component, approximately 30,000 individuals were sampled in each wave, and 2957 samples were previously diagnosed with diabetes. The proportion of excluded cases was small relative to the overall sample (see Supplementary Table 1).

### Outcomes and variable definition

The primary outcome focused on diabetes care performance across three domains: behavioral, clinical, and laboratory indicators. The thresholds used were based on several targets provided by the guidelines, including Indonesia's latest diabetes guideline and the American Diabetes Association 2025 guideline (See Supplementary Table 2 for details).^14^^,^^15^ Behavioral indicators included (B1) linkage to diabetes care defined as the use of antidiabetic treatment, (B2) Fiber intake (at least five portions of fruit and vegetables daily), (B3) physical activity (150 MET-minutes per week), and (B4) cessation from smoking. Clinical indicators included (C1) blood pressure control (systolic <140 mmHg and diastolic <90 mmHg), (C2) body mass index (BMI) within the normal range (18.5–22.9 kg/m^2^), and (C3) absence of central obesity, defined as waist circumference <90 cm for men and <80 cm for women. Laboratory indicators addressed metabolic control, including (L1) Satisfactory glycemic control, defined by fasting plasma glucose <130 mg/dL or HbA1c <7% for 2023. Lipid control included (L2) LDL-C <100 mg/dL, (L3) HDL-C ≥40 mg/dL for men and ≥50 mg/dL for women, (L4) Triglycerides <150 mg/dL, and (L5) total cholesterol <200 mg/dL. And (L6) Kidney function estimated by estimated glomerular filtration rate (eGFR) > 90 mL.

We also calculate composite targets, which serve as summary indicators, providing a more comprehensive assessment of how well individuals manage their condition across multiple relevant aspects. The behavioral-clinical composite score summed the number of satisfactory targets across four behavioral indicators and three clinical indicators, with a possible score from 0 to 7. The laboratory composite score included six targets ranging from 0 to 6. We also calculate the A-B-C target, which is the main target that is commonly used in previous studies, called A as glycemic control, B as blood pressure control, and C as LDL-C control.

Independent variables include survey year (2013, 2018, 2023), age group (15–39, 40–59, ≥60 years), gender, and domicile (urban vs. rural). Socioeconomic and care access indicators comprised education level, occupation, health insurance status, access to a car, and household wealth quintile. Stratified analyses were conducted to assess behavioral and clinical outcomes by province.

### Statistical analysis

Descriptive statistics were used to summarize the study population's demographic, socioeconomic, behavioral, clinical, and laboratory characteristics across the 2013, 2018, and 2023 surveys. Proportions and means were reported with 95% confidence intervals for each outcome domain, accounting for the complex survey design, including sampling weights, strata, and primary sampling units (PSUs). Province-level averages of behavioral-clinical composite scores were calculated to illustrate geographic patterns in diabetes care quality. Associations between independent variables and diabetes care targets were examined using survey-adjusted multilevel logistic regression models for behavioral, clinical, and laboratory outcomes, incorporating random intercepts at the province and island levels to account for geographic clustering (see equation in Supplementary Text 2. Model Equation). Education and occupation were excluded from laboratory outcomes to improve model stability due to smaller sample sizes. Composite scores were analyzed using survey-adjusted mixed-effects linear regression models with province and island as random effects. For regression models, adjusted odds ratios (AORs), beta coefficients, P-values, and 95% confidence intervals were reported, with p-values obtained directly from the survey-weighted regression estimates. All analyses were performed using R version 4.2.

A sensitivity analysis incorporating borderline states, values near but not meeting optimal targets, who may benefit from improved care, was conducted to identify low-hanging at-risk populations. All borderline criteria are detailed in Supplementary Table 2.

### Ethical approval

This study used de-identified, publicly available secondary data from the 2013, 2018, and 2023 Indonesian National Health Research (RISKESDAS and SKI) surveys. Ethical approval for this secondary analysis was obtained from the Harvard Institutional Review Board (Approval No: IRB23-1673/2024). As this study involved only anonymized data, no additional consent from participants was required.

### Role of the funding source

The authors did not receive any specific funding in conducting this study. The corresponding authors had full access to all the data and had final responsibility for the decision to submit for publication.

## Results

### Participants’ characteristics

The unweighted participants mean age increased over time in behavioral-clinical and laboratory samples, reaching 55.4 years overall in the behavioral-clinical group and 53.4 years in the laboratory group (Table 1). Women represented approximately 60.5% of the behavioral-clinical sample and 64.3% of the laboratory sample. Most participants resided in urban areas, and the wealthier population, where secondary education was the most common level attained. Across both groups, participant characteristics were broadly similar and stable over time, with consistent distributions in age, gender, domicile, education, occupation, and wealth. The percentage of insured individuals increased from 61.6% to 76.4% (details of sample characteristics provided in Supplementary Tables 3, 4, and 5).

**Table 1:** Characteristics of unweighted sample with prior diabetes diagnosis from Indonesia health survey 2013–2023.

Variable	Behavior and clinical sample				Laboratory sample			
	2013	2018	2023	Overall	2013	2018	2023	Overall
	(N = 13,158)	(N = 13,659)	(N = 15,407)	(N = 42,224)	(N = 931)	(N = 1055)	(N = 971)	(N = 2957)
**Age**								
Mean (SD)	54.1 (11.7)	55.4 (10.9)	56.5 (10.6)	55.4 (11.1)	51.6 (12.4)	53.7 (11.8)	54.7 (11.4)	53.4 (11.9)
Median [Min, Max]	54.0 [15.0, 98.0]	55.0 [15.0, 97.0]	57.0 [15.0, 98.0]	55.0 [15.0, 98.0]	52.0 [15.0, 86.0]	54.0 [15.0, 90.0]	55.0 [15.0, 86.0]	54.0 [15.0, 90.0]
**Sex**								
Female	7509 (57.1%)	8491 (62.2%)	9539 (61.9%)	25,539 (60.5%)	565 (60.7%)	681 (64.5%)	656 (67.6%)	1902 (64.3%)
Male	5649 (42.9%)	5168 (37.8%)	5868 (38.1%)	16,685 (39.5%)	366 (39.3%)	374 (35.5%)	315 (32.4%)	1055 (35.7%)
**Domicile**								
Rural	4815 (36.6%)	5460 (40.0%)	5085 (33.0%)	15,360 (36.4%)	379 (40.7%)	382 (36.2%)	338 (34.8%)	1099 (37.2%)
Urban	8343 (63.4%)	8199 (60.0%)	10,322 (67.0%)	26,864 (63.6%)	552 (59.3%)	673 (63.8%)	633 (65.2%)	1858 (62.8%)
**Education**								
Unschooled	2631 (20.0%)	3260 (23.9%)	2058 (13.4%)	7949 (18.8%)	198 (21.3%)	307 (29.1%)	124 (12.8%)	629 (21.3%)
Primary school	4003 (30.4%)	3713 (27.2%)	4530 (29.4%)	12,246 (29.0%)	306 (32.9%)	284 (26.9%)	330 (34.0%)	920 (31.1%)
Secondary school	4955 (37.7%)	5015 (36.7%)	6681 (43.4%)	16,651 (39.4%)	353 (37.9%)	374 (35.5%)	420 (43.3%)	1147 (38.8%)
Tertiary school	1569 (11.9%)	1671 (12.2%)	2138 (13.9%)	5378 (12.7%)	74 (7.9%)	90 (8.5%)	97 (10.0%)	261 (8.8%)
**Occupation**								
Unemployed	6186 (47.0%)	5979 (43.8%)	6220 (40.4%)	18,385 (43.5%)	424 (45.5%)	501 (47.5%)	389 (40.1%)	1314 (44.4%)
Informal sector	2209 (16.8%)	2660 (19.5%)	2908 (18.9%)	7777 (18.4%)	184 (19.8%)	221 (20.9%)	212 (21.8%)	617 (20.9%)
Formal sector	1935 (14.7%)	1603 (11.7%)	2092 (13.6%)	5630 (13.3%)	111 (11.9%)	92 (8.7%)	106 (10.9%)	309 (10.4%)
Private sector	2223 (16.9%)	2352 (17.2%)	2454 (15.9%)	7029 (16.6%)	160 (17.2%)	174 (16.5%)	156 (16.1%)	490 (16.6%)
Others	605 (4.6%)	1065 (7.8%)	1733 (11.2%)	3403 (8.1%)	52 (5.6%)	67 (6.4%)	108 (11.1%)	227 (7.7%)
**Wealth Quintile**								
1 (Poorest)	852 (6.5%)	1362 (10.0%)	1904 (12.4%)	4118 (9.8%)	69 (7.4%)	146 (13.8%)	142 (14.6%)	357 (12.1%)
2	1583 (12.0%)	1793 (13.1%)	2477 (16.1%)	5853 (13.9%)	125 (13.4%)	141 (13.4%)	187 (19.3%)	453 (15.3%)
3	2374 (18.0%)	2257 (16.5%)	2964 (19.2%)	7595 (18.0%)	209 (22.4%)	167 (15.8%)	207 (21.3%)	583 (19.7%)
4	3551 (27.0%)	3086 (22.6%)	3509 (22.8%)	10,146 (24.0%)	266 (28.6%)	230 (21.8%)	204 (21.0%)	700 (23.7%)
5 (Wealthiest)	4798 (36.5%)	5161 (37.8%)	4553 (29.6%)	14,512 (34.4%)	262 (28.1%)	371 (35.2%)	231 (23.8%)	864 (29.2%)
**Insurance status**								
No insurance	5057 (38.4%)	2948 (21.6%)	1980 (12.9%)	9985 (23.6%)	412 (44.3%)	267 (25.3%)	169 (17.4%)	848 (28.7%)
Have insurance	8101 (61.6%)	10,711 (78.4%)	13,427 (87.1%)	32,239 (76.4%)	519 (55.7%)	788 (74.7%)	802 (82.6%)	2109 (71.3%)

### Evaluation of diabetes behavioral target

**Fig. 1:**
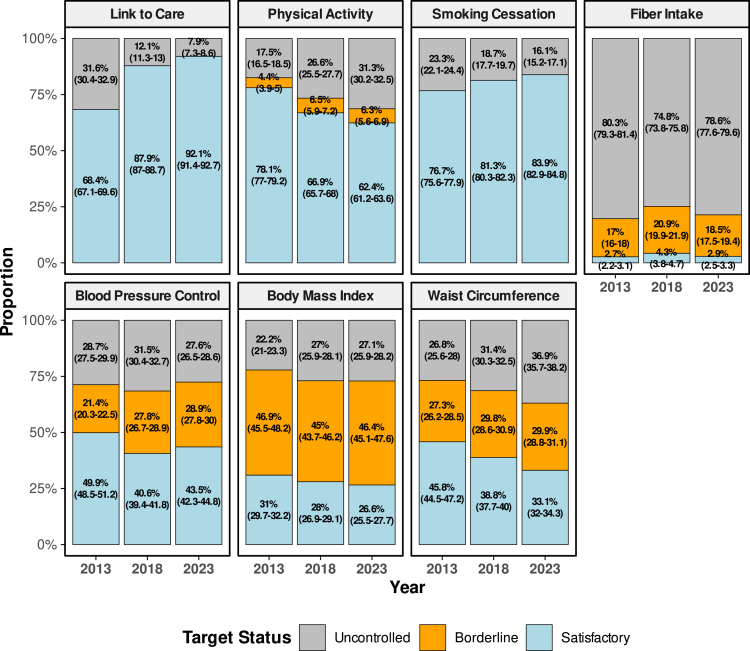
**The proportion of diagnosed diabetes population achieving behavioral and clinical targets, 2013–2023**. The figure displays weighted proportions of individuals with diagnosed diabetes achieving satisfactory, borderline, or uncontrolled thresholds for behavioral and clinical indicators. **Physical Activity**: Satisfactory if ≥ 500 MET-min/week; borderline for 150–499 MET-min/week; uncontrolled if < 150 MET-min/week. **Quit Smoking**: Satisfactory if not currently smoking (non-smoker or former smoker); uncontrolled if currently smoking. **Fiber Intake**: Satisfactory if > 5 portions per week; borderline if 3–5 portions; uncontrolled if ≤ 3 portions. **Blood Pressure Control**: Satisfactory if systolic <140 mmHg and diastolic <90 mmHg; borderline if only one is below threshold; uncontrolled if both are elevated. **BMI Level**: Satisfactory for BMI between 18.5 and 22.9 kg/m^2^; borderline if < 18.5 or 23–27.4 kg/m^2^; uncontrolled if ≥ 27.5 kg/m^2^. **Waist Circumference**: Satisfactory if < 90 cm (men) or <80 cm (women); borderline if 90–100 cm (men) or 80–90 cm (women); uncontrolled if above those thresholds. All estimates include 95% confidence intervals. Sample number: 42,195 adults with diagnosed diabetes in 2013, 2018, and 2023.

The proportion of participants with satisfactory behavioral and clinical characteristics is described in Fig. 1. Between 2013 and 2018, the proportion of the diagnosed diabetes population who were linked to care increased significantly from 68.4% (95% Confidence Interval: 67.1–69.6 to 92.1% (91.4–92.7%) in 2023. However, those who met the physical activity target declined from 78.1% (77.0–79.2%) in 2013 to 62.4% (61.2–63.6%) in 2023. Smoking abstinence improved steadily, from 76.1% (77.0–79.2%) in 2013 to 83.9% (82.9–84.8%) in 2023. The proportion of participants with satisfactory fiber intake showed poor control and minimal improvement, from 2.7% (2.2–3.1%) in 2013 to 2.9% (2.5–3.3%) in 2023. Overall, only those linked to care and smoking behavior showed improved performance between 2013 and 2023, while there was a marked decline in the proportion of subjects achieving satisfactory physical activity targets and a very low proportion of those with satisfactory fiber intake.

### Evaluation of diabetes clinical targets

We next analyzed the proportion of DM populations achieving satisfactory clinical targets that include blood pressure <140/90, BMI less than 23, and waist circumference <90 cm (male) or < 80 cm (female). The proportion of patients achieving blood pressure target was highest in 2013 at 49.9% (95% CI: 48.5–51.2%) but decreased to 40.6% in 2018 and slightly rose to 43.5% (42.3–44.8%) by 2023 (Fig. 1). The prevalence of DM people achieving satisfactory BMI declined steadily, from 31.2% (29.9–32.4%) in 2013 to 26.7% (25.6–27.9%) in 2023, with a corresponding increase in the proportion of people classified as uncontrolled BMI. In keeping with BMI data, the proportion of individuals with satisfactory waist circumference also fell from 45.8% (44.5–47.2%) to 33.1% (32.0–34.3%) over the same period. Overall, the analysis of clinical targets for diabetes care in Indonesia showed a worsening performance between 2013 and 2023.

### Evaluation of diabetes laboratory parameters

**Fig. 2:**
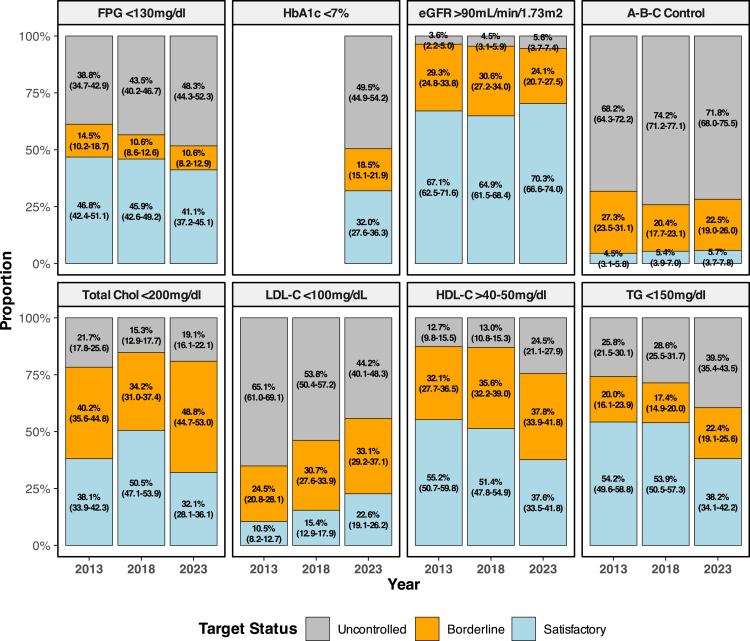
**The proportion of diagnosed diabetes population achieving laboratory targets, 2013–2023**. The figure presents weighted proportions of individuals with diagnosed diabetes achieving satisfactory, borderline, or uncontrolled thresholds across key laboratory indicators. **FPG:** Satisfactory control is defined as fasting plasma glucose (FPG) < 130 mg/dL, borderline FPG 130–150 md/dL; uncontrolled FPG>150 mg/dL. **HbA1c**: Satisfactory <7%, borderline 7–8.9%, uncontrolled ≥9%. **eGFR**: Satisfactory = Stage 1 (≥90 mL/min/1.73 m^2^); borderline = Stage 2–3a; uncontrolled = Stage 3b–5. **Total Cholesterol**: Satisfactory <200 mg/dL, borderline 200–250 mg/dL, uncontrolled >250 mg/dL. **LDL-C**: Satisfactory <100 mg/dL, borderline 100–130 mg/dL, uncontrolled >130 mg/dL. **HDL-C**: Satisfactory ≥40 mg/dL (men) or ≥50 mg/dL (women); borderline = 30–39 (men) or 40–49 (women); uncontrolled = <30 (men) or <40 (women). **Triglycerides**: Satisfactory <150 mg/dL; borderline 150–199 mg/dL; uncontrolled ≥200 mg/dL. **A-B-C Composite Control**: Satisfactory = simultaneous control of glucose (FPG), blood pressure <140 mmHg, and LDL-C <100 mg/dL; borderline = two out of three; uncontrolled = fewer than two. Confidence intervals are shown for each proportion. Sample number: 2953 in 2013, 2764 in 2018, and 2764 in 2023.

Analysis of laboratory indicators, including blood glucose and lipid profile in DM individuals, indicated no improvement between 2013 and 2023 (Fig. 2). The proportion of participants achieving satisfactory FPG levels declined from 30.4% (95% CI: 26.7–34.1%) in 2013 to 25.2% (21.6–28.8%) in 2023. Analysis of serum HbA1c levels, which was introduced in SKI 2023, indicated that only 32.0% (27.6–36.3%) of participants achieved satisfactory levels. Equally important, controlling serum lipid profile remained a persistent challenge for DM patients in Indonesia. Although the prevalence of subjects with satisfactory LDL-C (below 200 mg/dl) doubled from 10.5% (8.2–12.7%) in 2013 to 22.6% (19.1–26.2%) in 2023, HDL-C levels declined substantially, with only 37.6% (33.5–41.8%) meeting satisfactory levels by 2023. Consistently, subjects with satisfactory triglyceride levels decreased while cholesterol levels fluctuated. Kidney function, however, was generally better maintained (Fig. 2).

A sensitivity analysis was conducted to examine the proportion of individuals with diabetes who fell into a borderline category. In 2023, the highest borderline proportion was observed for total cholesterol, at 48.9% (95% CI: 44.7–53.0%), followed closely by body mass index, at 46.4% (45.1–47.6%). Other indicators with substantial borderline proportions included HDL cholesterol, at 37.8% (33.9–41.8%); waist circumference, at 29.9% (28.8–31.1%); and blood pressure, at 28.9% (27.8–30.0%).

### Proportion of participants achieving composite targets

**Fig. 3:**
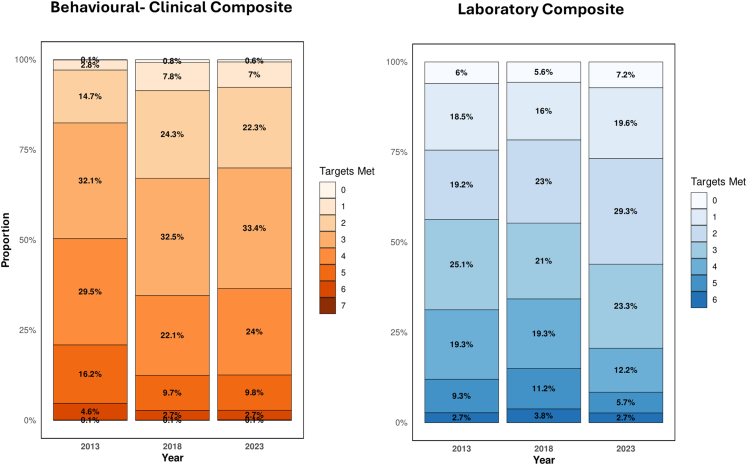
**Progress on composite diabetes management targets in Indonesia (2013–2023)**. Stacked bar charts showing the proportion of diagnosed diabetes patients meeting composite behavioral/clinical targets (left) and laboratory targets (right) in 2013, 2018, and 2023. Performance is grouped by the number of recommended targets met (0–7 for behavioral/clinical, 0 to 6 for laboratory). Color intensity increases with higher target achievement, indicating improved diabetes management.

As shown in Fig. 3 and Supplementary Figure 2, the overall performance in terms of achieving composite targets of diabetes management remained low across all years. Regarding the behavioral–clinical targets, the mean composite score (range 0–7) declined from 3.56 (95% CI: 3.53–3.59) in 2013 to 3.14 (3.11–3.17) in 2023, corresponding to an average change of −0.42 points (95% CI: −0.46 to −0.37, P < 0.001) (Fig. 3, Supplementary Table 6). And make only 2.7% (95% CI: 2.2–3.1) of participants who achieved all seven targets in 2023 For laboratory targets, the mean composite score (range 0–6) decreased from 2.72 (95% CI: 2.58–2.86) in 2013 to 2.42 (2.29–2.55) in 2023, an average decline of −0.30 points (95% CI: −0.50 to −0.11, P = 0.002) (Supplementary Table 7).

**Fig. 4:**
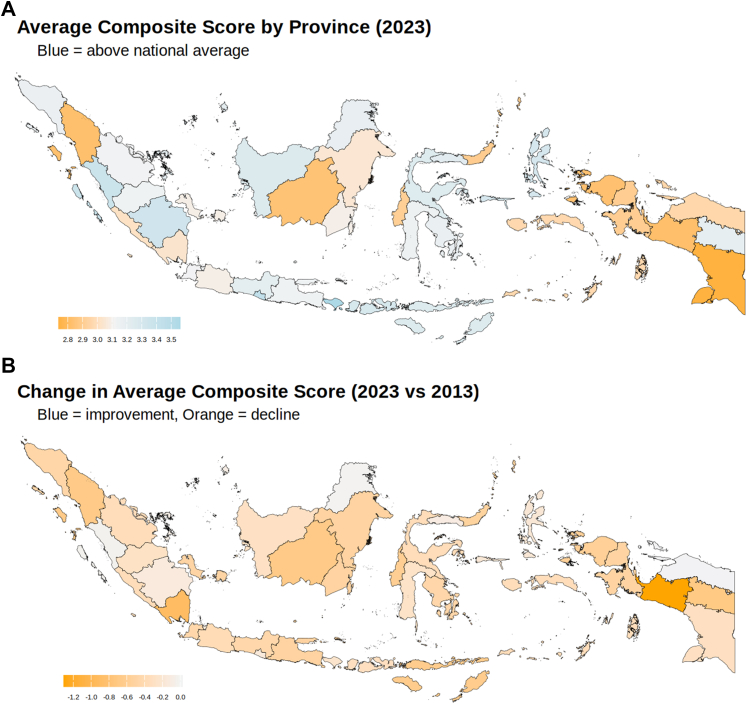
**Geographic Distribution and Trends in Composite Diabetes Management Scores (2023 vs. 2013)**. **A)** The average provincial composite score for diabetes behavioral and clinical management in 2023, with blue indicating provinces above the national average. **B)** The change in provincial mean scores from 2013 to 2023. Blue shades indicate improvement, while orange shades reflect a decline in average scores over the decade.

We performed geographical analysis of DM control performance across provinces in Indonesia by comparing behavioral composite scores in 2023 vs. 2013 (Fig. 4 and Supplementary Table 6). Overall, mean behavioral–clinical composite scores declined in nearly all provinces, with the largest declines observed in Papua Tengah (−1.31 points, 95% CI: −1.85 to −0.77), Lampung with −0.90 (−1.17 to −0.64), and no province showed improvement between 2013 and 2023. In 2023, Bali had the highest average behavioral-clinical composite score at 3.56 (3.39–3.73) while Papua Selatan recorded the lowest score at 2.74 (2.33–3.15).

### Factors associated with performance disparities

The decrease in target achievement by year was confirmed through multilevel logistic regression (Table 2). We also identified key individual and structural factors associated with achieving diabetes management targets. For behavioral outcomes, older adults were likelier to meet non-smoking and fiber intake goals but less likely to achieve physical activity targets. Men had significantly lower odds of achieving behavioral targets, especially for fiber intake (AOR: 0.02, 95% CI: 0.02–0.03) and physical activity (AOR: 0.69, 0.65–0.73). Tertiary education and wealth were strongly associated with better dietary and activity outcomes, but poorer obesity-related outcomes.

**Table 2:** Multilevel logistic regression assessing factors influencing the achievement of behavioral and clinical target for diagnosed diabetes in 2013–2023.

Predictors	Behavioral target–AOR (95% CI)				Clinical target–AOR (95% CI)			Composite target
	On treatment	Non-smoker	Fiber intake	Physical activity	Blood pressure	BMI	Central obesity	Beta estimate (95% CI)
**Intercept**	0.73 ∗∗(0.63–0.85)	23.49 ∗∗(19.1–28.93)	0.09 ∗∗(0.07–0.13)	6.37 ∗∗(5.27–7.70)	3.35 ∗∗(2.81–4.00)	3.79 ∗∗(3.08–4.66)	0.95 (0.78–1.14)	3.59 ∗∗∗ (3.48–3.69)
**Year (Survey)**								
2013	Ref	Ref	Ref	Ref	Ref	Ref	Ref	Ref
2018	3.54 ∗∗(3.31–3.79)	1.22 ∗∗(1.13–1.31)	1.43 ∗∗(1.34–1.52)	0.61 ∗∗(0.57–0.65)	0.94 ∗ (0.89–1.00)	0.81 ∗∗(0.76–0.86)	0.77 ∗∗(0.73–0.81)	−0.45 ∗∗∗ (−0.48 to −0.43)
2023	4.92 ∗∗(4.58–5.30)	1.47 ∗∗(1.36–1.59)	1.11 ∗∗(1.05–1.19)	0.42 ∗∗(0.39–0.44)	1.07 ∗ (1.01–1.13)	0.77 ∗∗(0.73–0.82)	0.62 ∗∗(0.59–0.66)	−0.42 ∗∗∗ (−0.45 to −0.39)
**Age group**								
15–40	Ref	Ref	Ref	Ref	Ref	Ref	Ref	Ref
41–60	2.20 ∗∗(2.02–2.39)	1.57 ∗∗(1.40–1.76)	1.19 ∗∗(1.08–1.31)	0.65 ∗∗(0.58–0.72)	0.47 ∗∗(0.43–0.52)	1.28 ∗∗(1.18–1.39)	0.78 ∗∗(0.73–0.85)	−0.08 ∗∗∗ (−0.12 to −0.04)
>60	2.48 ∗∗(2.25–2.74)	2.94 ∗∗(2.60–3.33)	1.21 ∗∗(1.09–1.34)	0.28 ∗∗(0.25–0.31)	0.50 ∗∗(0.45–0.55)	1.81 ∗∗(1.65–1.98)	0.84 ∗∗(0.77–0.91)	−0.15 ∗∗∗ (−0.20 to −0.11)
**Sex**								
Female	Ref	Ref	Ref	Ref	Ref	Ref	Ref	Ref
Male	0.78 ∗∗(0.74–0.83)	0.02 ∗∗(0.02–0.03)	0.82 ∗∗(0.78–0.87)	0.69 ∗∗(0.65–0.73)	1.27 ∗∗(1.20–1.33)	1.82 ∗∗(1.72–1.92)	4.40 ∗∗(4.19–4.63)	−0.05 ∗∗ (−0.07 to −0.02)
**Domicile**								
Rural	Ref	Ref	Ref	Ref	Ref	Ref	Ref	Ref
Urban	1.05 (0.99–1.12)	0.99 (0.92–1.06)	1.05 (0.99–1.12)	0.88 ∗∗(0.83–0.92)	1.05 ∗ (1.00–1.11)	0.90 ∗∗(0.85–0.95)	0.82 ∗∗(0.78–0.86)	−0.08 ∗∗∗ (−0.10 to −0.05)
**Education**								
Unschooled	Ref	Ref	Ref	Ref	Ref	Ref	Ref	Ref
Primary school	1.16 ∗∗(1.07–1.26)	1.06 (0.96–1.17)	1.24 ∗∗(1.14–1.34)	1.13 ∗∗(1.05–1.21)	1.03 (0.97–1.10)	0.98 (0.91–1.05)	0.91 ∗∗ (0.86–0.97)	0.05 ∗∗ (0.02–0.09)
Secondary school	1.10 ∗ (1.01–1.20)	1.21 ∗∗(1.09–1.33)	1.67 ∗∗(1.55–1.81)	1.33 ∗∗(1.24–1.43)	1.14 ∗∗(1.07–1.22)	0.84 ∗∗(0.78–0.91)	0.82 ∗∗(0.77–0.88)	0.09 ∗∗∗ (0.06–0.13)
Tertiary school	1.21 ∗∗ (1.07–1.37)	1.96 ∗∗(1.72–2.24)	2.14 ∗∗(1.93–2.38)	1.31 ∗∗(1.19–1.45)	1.22 ∗∗(1.11–1.34)	0.78 ∗∗(0.70–0.86)	0.72 ∗∗(0.65–0.79)	0.16 ∗∗∗ (0.11–0.21)
**Occupation**								
Unemployed	Ref	Ref	Ref	Ref	Ref	Ref	Ref	Ref
Informal sector	0.86 ∗∗(0.79–0.93)	0.55 ∗∗(0.50–0.61)	1.06 (0.99–1.15)	2.87 ∗∗(2.65–3.10)	1.23 ∗∗(1.15–1.32)	1.21 ∗∗(1.12–1.30)	1.40 ∗∗(1.31–1.49)	0.18 ∗∗∗ (0.14–0.21)
Formal sector	0.92 (0.83–1.02)	0.72 ∗∗(0.64–0.80)	1.07 (0.98–1.16)	1.76 ∗∗(1.61–1.92)	1.05 (0.97–1.14)	0.92 (0.85–1.00)	1.05 (0.97–1.13)	0.03 (−0.01–0.08)
Private sector	0.95 (0.87–1.03)	0.67 ∗∗(0.61–0.74)	1.13 ∗∗ (1.05–1.21)	1.86 ∗∗(1.73–2.00)	1.12 ∗∗ (1.05–1.19)	0.79 ∗∗(0.74–0.85)	0.91 ∗∗ (0.85–0.97)	0.01 (−0.02–0.05)
Others	0.88 ∗ (0.78–0.98)	0.76 ∗∗(0.66–0.86)	1.05 (0.96–1.16)	1.54 ∗∗(1.41–1.68)	1.21 ∗∗(1.12–1.32)	0.97 (0.89–1.06)	1.01 (0.93–1.10)	0.06 ∗∗ (0.02–0.11)
**Wealth quintile**								
1 (Poorest)	Ref	Ref	Ref	Ref	Ref	Ref	Ref	Ref
2	1.14 ∗ (1.02–1.27)	1.04 (0.91–1.18)	1.01 (0.91–1.13)	1.11 ∗ (1.01–1.22)	0.94 (0.86–1.03)	0.81 ∗∗(0.72–0.90)	0.83 ∗∗(0.76–0.91)	−0.05 ∗ (−0.10 to −0.00)
3	1.30 ∗∗(1.17–1.45)	1.11 (0.98–1.26)	1.15 ∗∗ (1.04–1.28)	1.08 (0.99–1.19)	0.99 (0.91–1.08)	0.72 ∗∗(0.65–0.79)	0.73 ∗∗(0.67–0.79)	−0.07 ∗∗ (−0.12 to −0.02)
4	1.50 ∗∗(1.35–1.67)	1.16 ∗ (1.03–1.32)	1.30 ∗∗(1.18–1.44)	1.13 ∗∗ (1.03–1.24)	0.99 (0.91–1.08)	0.66 ∗∗(0.60–0.73)	0.64 ∗∗(0.59–0.69)	−0.08 ∗∗∗ (−0.13 to −0.04)
5 (Wealthiest)	1.57 ∗∗(1.41–1.76)	1.33 ∗∗(1.17–1.50)	1.71 ∗∗(1.54–1.90)	1.12 ∗ (1.02–1.23)	1.08 (0.99–1.17)	0.56 ∗∗(0.51–0.62)	0.57 ∗∗(0.52–0.62)	−0.08 ∗∗ (−0.13 to −0.03)
**Insurance status**								
No insurance	Ref	Ref	Ref	Ref	Ref	Ref	Ref	Ref
Have insurance	1.14 ∗∗(1.07–1.22)	1.18 ∗∗(1.10–1.28)	1.03 (0.97–1.10)	0.95 (0.90–1.01)	1.07 ∗ (1.01–1.12)	0.97 (0.91–1.02)	0.98 (0.93–1.03)	0.07 ∗∗∗ (0.04–0.10)
**Random effects**								
σ^2^	3.29	3.29	3.29	3.29	3.29	3.29	3.29	1.38
τ_00_ Province	0.02	0.04	0.15	0.03	0.02	0.03	0.02	0.01
τ_00_ Island	0.00	0.01	0.09	0.02	0.02	0.03	0.03	0.01
ICC	0.01	0.01	0.07	0.01	0.01	0.02	0.02	0.01
N Province	38	38	38	38	38	38	38	38
N Island	6	6	6	6	6	6	6	6

∗P < 0.05, ∗∗P < 0.01

For laboratory targets, older age consistently predicted lower odds of achieving glycemic, lipid, and renal function goals (Table 3). Men were likelier to meet lipid targets but less likely to meet kidney function targets (AOR: 0.72, 95% CI: 0.60–0.85). Urban residence and higher wealth were also associated with lower odds of laboratory target achievement. Education and insurance showed limited associations with laboratory outcomes. Laboratory composite scores declined with increasing age and were lower among urban residents, while men had significantly higher laboratory composite scores (beta estimate 0.43 [95% 0.32–0.54]).

**Table 3:** Multilevel logistic regression assessing factors influencing the achievement of laboratory targets for diagnosed diabetes in 2013–2023.

Predictors	Laboratory target–AOR (95% CI)						ABC target^a^	Composite lab target
	Glycemic control (A)	Cholesterol	LDL	HDL	Triglycerides	CKD Lab	AOR (95% CI)	Beta estimate (95% CI)
**Intercept**	3.94 ∗∗ (2.79–5.58)	11.46 ∗∗(6.86–19.14)	1.05 (0.74–1.50)	4.22 ∗∗(2.67–6.66)	6.17 ∗∗(4.12–9.24)	16.42 ∗∗(10.27–26.26)	0.33 ∗∗(0.18–0.60)	3.80 ∗∗∗ (3.56–4.03)
**Year (Survey)**								
2013	Ref	Ref	Ref	Ref	Ref	Ref	Ref	Ref
2018	0.91 (0.75–1.09)	1.26 (0.98–1.62)	1.40 ∗∗ (1.14–1.71)	0.78 (0.59–1.04)	0.79 ∗ (0.64–0.99)	1.01 (0.82–1.25)	0.85 (0.53–1.36)	−0.02 (−0.15–0.12)
2023	0.71 ∗∗(0.59–0.87)	1.04 (0.81–1.33)	2.06 ∗∗(1.67–2.53)	0.42 ∗∗(0.32–0.54)	0.47 ∗∗(0.38–0.58)	1.22 (0.98–1.52)	0.87 (0.53–1.43)	−0.45 ∗∗∗ (−0.59 to −0.31)
**Age group**								
15–40	Ref	Ref	Ref	Ref	Ref	Ref	Ref	Ref
41–60	0.36 ∗∗(0.28–0.46)	0.36 ∗∗(0.25–0.52)	0.53 ∗∗(0.42–0.67)	1.09 (0.80–1.47)	0.54 ∗∗(0.41–0.72)	0.27 ∗∗(0.18–0.39)	0.19 ∗∗(0.12–0.28)	−0.87 ∗∗∗ (−1.03 to −0.71)
>60	0.47 ∗∗(0.36–0.61)	0.43 ∗∗(0.29–0.65)	0.55 ∗∗(0.42–0.71)	1.25 (0.89–1.76)	0.69 ∗ (0.51–0.94)	0.10 ∗∗(0.07–0.15)	0.11 ∗∗(0.06–0.20)	−0.88 ∗∗∗ (−1.05 to −0.70)
**Sex**								
Female	Ref	Ref	Ref	Ref	Ref	Ref	Ref	Ref
Male	1.46 ∗∗(1.25–1.71)	2.35 ∗∗(1.88–2.94)	1.63 ∗∗(1.39–1.92)	3.12 ∗∗(2.43–4.00)	1.13 (0.95–1.34)	0.72 ∗∗(0.60–0.85)	2.09 ∗∗(1.42–3.07)	0.43 ∗∗∗ (0.32–0.54)
**Domicile**								
Rural	Ref	Ref	Ref	Ref	Ref	Ref	Ref	Ref
Urban	0.80 ∗∗ (0.68–0.94)	0.79 ∗ (0.63–0.98)	0.89 (0.75–1.06)	1.02 (0.81–1.27)	1.12 (0.93–1.35)	0.82 ∗ (0.68–0.99)	0.66 ∗ (0.44–1.00)	−0.12 ∗ (−0.24 to −0.01)
**Wealth quintile**								
1 (Poorest)	Ref	Ref	Ref	Ref	Ref	Ref	Ref	Ref
2	0.78 (0.58–1.04)	0.78 (0.52–1.17)	0.80 (0.59–1.07)	0.93 (0.64–1.35)	0.93 (0.67–1.28)	0.96 (0.69–1.35)	0.42 ∗∗ (0.22–0.79)	−0.15 (−0.35–0.05)
3	0.68 ∗∗ (0.51–0.89)	0.49 ∗∗(0.34–0.72)	0.74 ∗ (0.56–0.98)	1.01 (0.70–1.44)	0.84 (0.62–1.14)	0.84 (0.61–1.15)	0.41 ∗∗ (0.22–0.74)	−0.23 ∗ (−0.43 to −0.04)
4	0.65 ∗∗ (0.50–0.86)	0.70 (0.48–1.03)	0.76 (0.58–1.01)	1.12 (0.78–1.60)	0.83 (0.61–1.12)	0.82 (0.60–1.12)	0.37 ∗∗ (0.20–0.68)	−0.31 ∗∗ (−0.49 to −0.12)
5 (Wealthiest)	0.59 ∗∗(0.45–0.78)	0.78 (0.53–1.14)	0.70 ∗ (0.53–0.93)	1.06 (0.74–1.51)	0.76 (0.56–1.02)	0.76 (0.56–1.03)	0.47 ∗ (0.26–0.84)	−0.39 ∗∗∗ (−0.58 to −0.21)
**Insurance status**								
No insurance	Ref	Ref	Ref	Ref	Ref	Ref	Ref	Ref
Have insurance	1.07 (0.90–1.27)	1.02 (0.82–1.28)	1.04 (0.87–1.25)	0.96 (0.76–1.22)	0.95 (0.78–1.15)	0.75 ∗∗ (0.62–0.92)	1.05 (0.68–1.61)	−0.10 (−0.22–0.02)
**Random effects**								
σ2	3.29	3.29	3.29	3.29	3.29	3.29	3.29	1.95
τ00 Province	0.00	0.04	0.05	0.11	0.03	0.00	0.00	0.01
ICC	0	0.01	0.02	0.03	0.01		0	0.01
N Province	37	37	37	37	37	37	37	37

∗P < 0.05, ∗∗P < 0.01 ∗∗∗, P < 0.001.

a ABC target stand for Satisfaction in Glycemic Control Target (FPG< 130mgDl), Blood Pressure Target (SBP <140 mmHg AND DBP<90 mmHg), and LDL-C Target (LDL <100mg/Dl).

In the multilevel model, between-province variance contributed minimally (ICC ≈ 0.01). By contrast, fiber intake (ICC = 0.07) and HDL-C (ICC = 0.03) showed relatively higher between-province variability, suggesting these factors may have a geographical dimension. The provincial random intercepts for behavioral-clinical composite scores show meaningful differences, with Bali, Yogyakarta, and Nusa Tenggara Timur having the highest positive deviations. In contrast, Papua, West Java, and South Sulawesi had the lowest (Supplementary Figure 3).

## Discussion

This study provides the most comprehensive longitudinal assessment of diabetes care performance in Indonesia to date, with detailed indicators of performance across decades and a large sample size representative of the national and provincial levels. Our main finding shows that treatment coverage was increased, while performance across domains is inadequate and worsens over the year. Only a small proportion of individuals with diabetes achieved satisfactory control in behavioral-clinical, laboratory, and composite outcomes, with all provinces in Indonesia scoring a decline over time. These findings are consistent with earlier studies that have documented fragmented diabetes care and low levels of glycemic control in Indonesia.^7^ And low adherence to recommended screening practices, such as annual HbA1c testing or lipid monitoring.^8^^,^^16^

Indonesia's diabetes care performance is comparable with India, Brazil, Mexico, Vietnam, and Malaysia, which shows that around ∼30–40% of DM populations achieve HbA1C Target.^17^, ^18^, ^19^, ^20^, ^21^ The performance of DM care in these countries is lower than that of developed countries like the US and Singapore.^13^^,^^22^^,^^23^ Colombia and China are examples of developing countries with the best performance in diabetes care, maintaining 48%–64% of their diagnosed diabetes population on the HbA1c target and 20% in the composite target.^24^^,^^25^ While contextual differences exist, the overall level of laboratory control and composite achievement in Indonesia, especially the <5% reaching all composite lab targets, is strikingly low. This gap highlights missed opportunities for implementing multi-component chronic care targets.

The huge increase in diabetes treatment between 2013 and 2018 was most likely due to the implementation of the national health insurance (JKN) in 2014, which helped to remove financial access barriers.^26^ However, the rise in patient treatment was not accompanied by improvements in performance, laboratory, and composite outcome measures, which plateaued or declined. One key structural factor is the availability and distribution of healthcare resources, which have not kept pace with the rising burden of diabetes. In 2022, Indonesia had 3.84 health workers per 1000 people on average, but with wide district variation (0.31–19.08). Physician distribution was skewed toward urban areas (1.10 GPs and 0.35 specialists per 1000) compared to rural areas (0.27 and 0.06, respectively).^27^ This maldistribution means that health resources do not align with the geographic spread of diabetes, which is shown to largely increase in rural areas. The COVID-19 pandemic further disrupted diabetes care in Indonesia, with a national survey showing that nearly 70% of people with diabetes faced difficulties accessing consultations, medications, monitoring, diet, or exercise.^28^

Anthropometric targets showed a clear decline, with worsening obesity and an increase in central obesity. The sharper rise in central obesity than general obesity raises concern that weight gain is increasingly driven by visceral fat accumulation, which carries greater cardiovascular risk than general obesity alone.^29^, ^30^, ^31^, ^32^ Unfortunately, central obesity is not a management target in Indonesia's national diabetes guidelines and is rarely assessed as a routine clinical parameter in primary care settings.^33^ Fiber intake showed the poorest status, with nearly 78% of individuals with diabetes reporting rarely consuming fruits or vegetables. Misconceptions about fructose in fruit, despite no such restriction in the national diabetes guidelines, may further suppress intake.^34^, ^35^, ^36^ The laboratory outcomes reflect a systemic shortfall in behavioral and clinical management. Trends in HDL-C and triglycerides are consistent with broader population shifts.^37^ LDL-C control emerged as an exception, with consistent improvement over time. This trend is also observed in Singapore, where HbA1c and blood pressure control fluctuated, but LDL consistently improved.^23^

The parallel decline in behavioral and clinical targets among people with diagnosed diabetes, together with similar patterns in the undiagnosed population (Supplementary Table 3), suggests that the problem stems not only from inadequate treatment but also from worsening health behaviors at the population level, which is likely shaped by rapid economic growth, urbanization, and increasing adoption of high-risk lifestyles. Beyond clinical management, population-level prevention strategies such as clear nutrition labeling on food and beverage products, social awareness campaigns across multiple platforms, and multi-setting interventions in schools, workplaces, and communities are essential as blanket policies to reduce baseline risk. While these interventions are not examined in detail in this study, they remain critical complements to clinical strategies for addressing the dual challenge of diabetes prevention and management.

Social determinants remain strongly associated with performance; for instance, tertiary education has been shown to double the odds of achieving treatment targets, yet only 11.7% of individuals with diabetes have completed higher education. Education and income gradients highlight how health literacy and socioeconomic position continue to shape access to care and the ability to act on health information.^38^ Low education combined with high income is linked to adverse health outcomes, as reported in China and other transitioning economies.^39^ Transition is also reflected in physical activity targets, where occupational and environmental influences: office workers are more sedentary than those in informal sectors, and urbanization has intensified this trend in a context where most Indonesian cities lack a walkable design. Indonesia has been identified as one of the least active countries globally, with very low average daily step counts.^40^ Smoking remains a highly gendered behavior, with Indonesian males reporting the highest smoking rates globally.^41^

Indonesia's wide cultural and geographic diversity likely influences both health behavior and clinical practice.^42^ In our individual-level multilevel models, we found that even after adjusting for socioeconomic status, provinces such as Jakarta and West Java had lower composite diabetes care scores, while Yogyakarta and Bali performed better despite comparable economic conditions. This suggests that factors beyond economics, such as cultural norms, local health system practices, or geographic context, might play an important role.^43^^,^^44^

### Implications for policy

Non-adherence and limited continuity of care among people with diabetes are influenced by multiple factors, including access to health facilities, side effects of treatment, financial constraints, and the presence of complications.^45^^,^^46^ In Indonesia, the implementation of national health insurance has primarily expanded coverage, but the performance of chronic disease management remains poor.^47^^,^^48^ Many primary care facilities still lack routine access to laboratory testing, which limits timely diagnosis and monitoring.^49^^,^^50^ In addition, the absence of structured follow-up mechanisms (i.e., use of the digital tools), referral feedback loops, or chronic care registries contributes to fragmented care and missed treatment intensification opportunities.^51^, ^52^, ^53^, ^54^ These structural deficiencies weaken disease control even when patients are engaged with the health system. On the provider side, primary care physicians may lack incentives or training to adhere to evidence-based chronic disease guidelines.^55^, ^56^, ^57^, ^58^ Clinicians may default to symptomatic management over long-term risk reduction strategies without adequate time, support tools, or accountability structures.

Community-based programs (such as posbindu) are often seen as a complement to primary healthcare for NCDs, but most evidence comes from short-term, small-scale, or observational studies. One robust evaluation found gains in preventive behaviors and knowledge, though consistent effects on metabolic outcomes were lacking.^59^^,^^60^ Given their wide reach, these programs can support primary care screening, but their impact is constrained by limited training, resources, and competing workloads.^61^

Lessons could be taken from China's approach, which uses a comprehensive structural approach to managing NCD.^62^ China's national success in diabetes control provides a compelling example of how structured policies can enhance chronic disease outcomes. Its Diabetes Prevention and Control Action Plan is rooted in the “3.3.3.1 strategy,” which addresses three target groups (the general population, high-risk individuals, and patients) across three dimensions: risk factor control, early screening and diagnosis, and chronic disease management by health promotion, targeted interventions, and structured disease management.^63^ In the Indonesian context, this practicality could be adopted by first revising existing regulations and national clinical guidelines to define the targeted population groups and standardized care pathways clearly. These updated guidelines should then be used to guide implementation. To enhance uptake, broad education and capacity-building efforts should be conducted for both health care providers and patients.

Improving diabetes outcomes in Indonesia will require shifting from episodic care to structured, continuous chronic disease management.^46^^,^^58^ Indonesia's diabetes guidelines, currently led by the Indonesian Society of Endocrinology (Perkeni), should also evolve to include composite indicators that better reflect the multifactorial nature of diabetes management. We note that many indicator targets remained the same from a decade ago. Adding composite metrics would promote more integrated care and offer more emphasis on managing multiple risk factors. In addition, a truly holistic approach should also recognize the role of mental health, given its influence on treatment adherence, self-management, and overall quality of life.^64^

The Lancet Diabetes Commission recommends LMICs transform the health-care ecosystem by moving beyond individual clinician efforts toward system-wide integration that enables early diagnosis, risk stratification, and tailored interventions.^65^ In Indonesia, this would require interoperable health information systems, standardized diabetes–CVD care pathways, reliable medicine and supply chains, and performance feedback at the district and facility levels to effectively close prevention and care gaps.^65^ Those efforts, including behavioral changes and treatment adherence, should be driven by government regulation policies as the leading enabler of behavior modification, while provider and community organizations such as the Indonesian Diabetes Educator Association (Pedi), the Indonesian Diabetes Association (Persadia), and Perkeni can help policy implementation and extend its impact at the community level.

### Strengths and limitations

This study has several strengths, including using nationally representative, repeated cross-sectional data spanning a decade, three survey waves, and multilevel modeling to capture individual and geographic variation. Including composite scores across domains offers a comprehensive assessment of diabetes care. However, the cross-sectional design limits causal inference, and smaller sample sizes for laboratory outcomes may reduce precision in subgroup analyses. Some clinical and complication data were based on self-reports. Survival bias may also influence results, as individuals with poorly managed or severe diabetes may be underrepresented, potentially leading to an overly optimistic view. In addition, the behavioral-clinical composite score assumes equal weighting of all indicators, which may not fully reflect their relative epidemiological importance and limits comparability. However, from a clinical and patient perspective, all targets remain important. further study should explore the weighted importance of each risk. Despite these limitations, the study offers robust, policy-relevant insights into diabetes care in Indonesia.

### Conclusion

Despite expanded coverage, diabetes care performance in Indonesia remains critically low, particularly in achieving composite metabolic and behavioral targets. Addressing this gap will require a shift toward integrated, outcome-based guidelines and more structured chronic disease management anchored in primary care.

## Contributors

FRM: Conceptualization, Investigation, Data Curation, Formal Analysis, Methodology, Visualization, Writing—Original Draft, And Project Administration.

JBS: Data Curation, Formal Analysis, Visualization, Project Administration.

DLT: Methodology, Investigation, Validation, Writing – review & editing.

SAN: Investigation, Validation, Writing – review & editing.

DO: Conceptualization, Methodology, Investigation, Writing – review & editing.

## Data sharing statement

The data used to support this study are available from the Data Management Laboratory of layanandata.kemkes.go.id by Indonesia's Ministry of Health on reasonable request with prior official written permission.

## Editor note

The Lancet Group takes a neutral position with respect to territorial claims in published maps and institutional affiliations.

## Declaration of interest

The authors declare that they have no conflicts of interest.
