# Supplementary material for: Diabetes care performance in Indonesia: a serial cross-sectional analysis of behavioral, clinical, and laboratory outcomes from 2013 to 2023
Source: Lancet Reg Health West Pac. 2025 Nov 23;65:101759. doi: 10.1016/j.lanwpc.2025.101759 (PMC12681878; doi:10.1016/j.lanwpc.2025.101759)
Supplement: Supplementary Material [file mmc1.pdf]

**SUPPLEMENTARY MATERIALS**

# Diabetes Care Performance in Indonesia: A Serial Cross-Sectional Analysis of Behavioral, Clinical, and Laboratory Outcomes From 2013 to 2023

Farizal Rizky Muharram[1,2,*], Julian Benedict Swannjo[2], Dicky L. Tahapary[3,4], Sally A. Nasution[5], Delvac Oceandy[6,*]

[1] Global Health and Social Medicine, Harvard Medical School, Boston, USA

[2] Health System Center, ARC Institute, Jakarta, Indonesia

[3] Division of Endocrinology, Metabolism, and Diabetes, Department of Internal Medicine. Faculty of Medicine Universitas Indonesia, Dr. Cipto Mangunkusumo Hospital, Jakarta, Indonesia

[4] Metabolic, Cardiovascular, and Aging Research Cluster. The Indonesian Medical Education and Research Institute, Faculty of Medicine, Universitas Indonesia.

[5] Department of Internal Medicine, Faculty of Medicine, Universitas Indonesia, Jakarta, Indonesia

[6] Division of Cardiovascular Sciences, Faculty of Biology, Medicine and Health, University of Manchester, United Kingdom.

**Supplementary Text 1 - 2**

**Supplementary Table 1 – 7**

**Supplementary Figure 1 - 3**

**Harmonization of Survey Questionnaire on Behavioral Target**

**Supplementary Text 1. Sampling, Weighting, and Response Rate of Indonesian National Survey**

Both Riskesdas (2013, 2018) and SKI (2023) used a complex, multistage stratified cluster sampling design based on random selection of census blocks (urban and rural strata) and households within each block. For each participant, the base weight was defined as the inverse of the selection probability across all sampling stages. These base weights were then adjusted for household and individual non-response, and post-stratified to match national demographic distributions from the corresponding year's population census. Survey weights were provided by the Indonesian National Institute of Health Research and Development (NIHRD) and the Ministry of Health's Center for Data and Information. We then made additional reweighting as diagnosed diabetes was overrepresented in the biomedical sample. To account for selection bias and population representativeness, reweighting has been performed by adjusting the biomedical sample weights to match the national survey sample, which represents the national population. The final weights ensure representativeness at the national (and for interview data, provincial) levels. Applying these weights allows us to obtain nationally representative estimates by correcting for unequal selection probabilities and differential response rates, ensuring that over- or under-represented subgroups contribute appropriately to the total estimates. This improves representativeness and reduces bias in population-level estimates.

Response rate data is provided in **Supplementary** Error! Reference source not found. **1.** For Riskesdas 2013, the individual survey achieved a response rate of 99.88% for census blocks, 98.32% for households, and 92.52% for individuals. The biomedical component had a 100.00% response rate for census blocks and a 54.27% response rate for households and individuals. In Riskesdas 2018, the individual survey had a response rate of 99.41% for census blocks, 98.57% for households, and 93.20% for individuals, while the biomedical survey showed a 99.92% response rate for census blocks, 77.73% for households, and 63.58% for individuals. SKI 2023 reported a 98.74% response rate for census blocks, 92.69% for households, and 81.03% for individuals in the individual survey, and the biomedical survey had a 97.72% response rate for census blocks, 72.66% for households, and 49.92% for individuals.

**Supplementary Text 2. Model Equation**

All analyses accounted for the complex survey design of Riskesdas 2013, Riskesdas 2018, and SKI 2023, including sampling weights ($w_{hip}$), strata (h), and primary sampling units (PSUs) (p, census blocks). Each respondent i belongs to PSU p, stratum h, province j, and island k. Survey weights were normalized within strata and incorporated into all descriptive and regression analyses.

**1. Multilevel Logistic Regression (binary outcome: target achieved)**

For individual i in province j, and island k

$$logit\{Pr(Y_{ijk} = 1)\} = \beta 0 + X_{ijk}^T \beta + u_j + v_k$$

where

- $Y_{ijk}$ = 1 if the diabetes management target is achieved, 0 otherwise.
- $X_{ijk}$ = vector of covariates (age, sex, education, wealth quintile, domicile, survey year, etc.).
- β0, β = fixed effects.
- $u_j \sim N(0, \sigma^2_u)$ = province random intercept.
- $v_k \sim N(0, \sigma^2_v)$ = island random intercept.

**2. Mixed-Effects Linear Regression (continuous outcome: composite scores)**

For individual i in province j, and island k (behaviour only):

$$S_{ipjk} = \beta 0 + X_{ijk}^T \beta + u_j + v_k + \varepsilon_{ijk}$$

where

- $S_{ijk}$ = behavioral–clinical composite score (0–7) or laboratory composite score (0–6).
- $\varepsilon_{ijk} \sim N(0, \sigma^2)$ = individual error term.
- β0, β = fixed effects.
- $u_j \sim N(0, \sigma^2_u)$ = province random intercept.
- $v_k \sim N(0, \sigma^2_v)$ = island random intercept.

Survey weights, strata, and PSU clustering were incorporated as in the logistic model.

## Supplementary Table 1. Response Rate of The Indonesia Health Survey in 2013, 2018 and 2023

| Survey | Census Block | | | Household | | | Individual | | |
|---|---|---|---|---|---|---|---|---|---|
| | Target | Response | Response Rate | Target | Response | Response Rate | Target | Response | Response Rate |
| **Riskesdas 2013** | | | | | | | | | |
| Individual | 12,000 | 11,986 | 99.88% | 300,000 | 294,959 | 98.32% | 1,027,763 | 950,933 | 92.52% |
| Biomedic | 1,000 | 1,000 | 100.00% | 92,000 | 49,931 | 54.27% | 92,000 | 49,931 | 54.27% |
| **Riskesdas 2018** | | | | | | | | | |
| Individual | 30,000 | 29,824 | 99.41% | 300,000 | 295,720 | 98.57% | 1,091,528 | 1,017,290 | 93.20% |
| Biomedic | 2,500 | 2,498 | 99.92% | 24,980 | 19,418 | 77.73% | 75,000 | 47,687 | 63.58% |
| **SKI 2023** | | | | | | | | | |
| Individual | 34,500 | 34,065 | 98.74% | 340,555 | 315,646 | 92.69% | 1,082,958 | 877,531 | 81.03% |
| Biomedic | 2,500 | 2,443 | 97.72% | 25,000 | 18,164 | 72.66% | 75,000 | 37,443 | 49.92% |

**Supplementary Table 2. Diabetes Management Performance Stage**

| Type | Outcome | Target State | Borderline State | Uncontrolled State |
|---|---|---|---|---|
| Behavior (B) | B1 Treated / Link to Care | Get treatment (routine) | Get treatment (not routine) | Not get treatment |
| | B2 Fiber Intake[5] | >5 portion | 3-5 portion | ≤3 portion |
| | B3 Physical Activity[1,3] | MET ≥150 | MET 100-150 | MET <100 |
| | B4 Quit Smoking[1,2] | Not Smoking | Smoking | Smoking |
| Clinical (C) | C1 Blood Pressure Control (SBP/DBP, mmHg)[1,2,*] | <140 & <90 | <140 / <90 | ≥140 & ≥90 |
| | C2 BMI Control (kg/m$^2$)[2,3] | 18.5 – 22.9 | <18.5 or 23-27.4 | ≥27.5 |
| | C3 Central Obesity Control[2,3] | <90 cm (male)<br><80 cm (female) | 90-100 cm (male)<br>80-90 cm (female) | ≥100 cm (male)<br>≥90 cm (female) |
| Laboratory (L) | L1 Glycemic Control (FPG mg/dL)[1,2] | FPG <130 | FPG 130-150 | FPG >150 |
| | L1 Glycemic Control ( HbA1c %)[1,2] | <7 | 7.0-8.9 | ≥9 |
| | L2 Total Cholesterol (mg/dl)[1,2,4] | <200 | 200-250 | ≥250 |
| | L3 LDL-C (mg/dl)[1,2,4] | <100 | 100-130 | ≥130 |
| | L4 HDL (mg/dl)[1,2,4] | ≥40 (male)<br>≥50 (female) | 30-39 (male)<br>40-49 (female) | <30 (male)<br><40 (female) |
| | L5 Triglycerides (mg/dl)[1,2,4] | <150 | 150-199 | ≥200 |

References:
1. ADA Standards of Medical Care in Diabetes. 2024
2. PERKENI Clinical Guidelines (Indonesia, 2021)
3. WHO/IDF Guidelines (BMI, waist circumference, physical activity for Asian populations)
4. NCEP ATP III Guidelines (lipids)
5. WHO Dietary Recommendations (≥400 g fruits/vegetables per day ≈ ≥5 portions/day), adapted in RISKESDAS questionnaire

* Blood pressure target of ADA was varied between years, while Indonesian Endocrinology Association Target remain the same in all survey year. For local target and comparability between year, we use SBP 140- and DBP 90 as target.

**Supplementary Table 3. Characteristics of participants who were diagnosed with DM and participants were not diagnosed with DM during Riskesdas 2013, 2018 and SKI 2023**

| Variable | 2013 | | 2018 | | 2023 | |
|---|---|---|---|---|---|---|
| | **Diagnosed** n = 931 | **Not Diagnosed** **n** = 2,909 | **Diagnosed** n = 1,055 | **Not Diagnosed** n = 2,720 | **Diagnosed** n = 971 | **Not Diagnosed** n = 2,000 |
| **On DM Treatment** | | | | | | |
| Not Treated | 363 (39%) | 2,909 (100%) | 253 (24%) | 2,720 (100%) | 184 (19%) | 2,000 (100%) |
| Treated | 568 (61%) | 0 (0%) | 802 (76%) | 0 (0%) | 787 (81%) | 0 (0%) |
| **Glycaemic Control** | | | | | | |
| Uncontrolled | 611 (66%) | 2,668 (92%) | 741 (70%) | 2,584 (95%) | 740 (76%) | 1,895 (95%) |
| Controlled | 320 (34%) | 241 (8.3%) | 314 (30%) | 136 (5.0%) | 231 (24%) | 105 (5.3%) |
| **Blood Pressure Control** | | | | | | |
| uncontrolled | 446 (48%) | 1,445 (50%) | 638 (61%) | 1,556 (57%) | 546 (57%) | 1,094 (55%) |
| controlled | 481 (52%) | 1,456 (50%) | 415 (39%) | 1,153 (43%) | 420 (43%) | 895 (45%) |
| Unknown | 4 | 8 | 2 | 11 | 5 | 11 |
| **BMI Status** | | | | | | |
| Underweight | 145 (16%) | 645 (22%) | 138 (13%) | 441 (16%) | 94 (9.8%) | 253 (13%) |
| Normal | 384 (42%) | 1,164 (40%) | 390 (37%) | 952 (35%) | 375 (39%) | 681 (34%) |
| Overweight | 280 (30%) | 766 (27%) | 347 (33%) | 850 (31%) | 334 (35%) | 667 (34%) |
| Obese Class I | 83 (9.0%) | 236 (8.2%) | 134 (13%) | 356 (13%) | 123 (13%) | 279 (14%) |
| Obese Class II | 32 (3.5%) | 74 (2.6%) | 37 (3.5%) | 101 (3.7%) | 34 (3.5%) | 96 (4.9%) |
| Unknown | 7 | 24 | 9 | 20 | 11 | 24 |
| **Waist Control** | | | | | | |
| uncontrolled | 477 (53%) | 1,237 (44%) | 627 (60%) | 1,420 (53%) | 583 (64%) | 1,168 (62%) |
| controlled | 426 (47%) | 1,606 (56%) | 413 (40%) | 1,260 (47%) | 335 (36%) | 723 (38%) |
| Unknown | 28 | 66 | 15 | 40 | 53 | 109 |
| **Cholesterol Control** | | | | | | |
| uncontrolled | 513 (59%) | 1,288 (47%) | 514 (52%) | 1,090 (43%) | 629 (70%) | 1,144 (61%) |
| controlled | 359 (41%) | 1,443 (53%) | 479 (48%) | 1,433 (57%) | 273 (30%) | 728 (39%) |
| Unknown | 59 | 178 | 62 | 197 | 69 | 128 |
| **LDL Control** | | | | | | |
| uncontrolled | 764 (88%) | 2,289 (84%) | 850 (86%) | 2,115 (84%) | 700 (78%) | 1,446 (77%) |
| controlled | 108 (12%) | 442 (16%) | 143 (14%) | 408 (16%) | 202 (22%) | 426 (23%) |
| Unknown | 59 | 178 | 62 | 197 | 69 | 128 |
| **HDL Control** | | | | | | |
| uncontrolled | 441 (47%) | 1,509 (52%) | 558 (53%) | 1,407 (52%) | 660 (68%) | 1,371 (69%) |
| controlled | 490 (53%) | 1,400 (48%) | 497 (47%) | 1,313 (48%) | 311 (32%) | 629 (31%) |
| **Fiber (Fruit + Vegetable) Intake** | | | | | | |
| Not Enough | 771 (97%) | 2,339 (98%) | 843 (96%) | 2,157 (96%) | 860 (97%) | 1,751 (97%) |
| Enough | 26 (3.3%) | 52 (2.2%) | 37 (4.2%) | 79 (3.5%) | 28 (3.2%) | 54 (3.0%) |
| Unknown | 134 | 518 | 175 | 484 | 83 | 195 |
| **Smoking Status** | | | | | | |
| Not Smoking | 738 (79%) | 2,190 (75%) | 890 (84%) | 2,202 (81%) | 843 (87%) | 1,648 (82%) |
| Smoking | 193 (21%) | 719 (25%) | 165 (16%) | 518 (19%) | 128 (13%) | 352 (18%) |
| **BC Control** | | | | | | |
| uncontrolled | 825 (91%) | 2,568 (91%) | 959 (93%) | 2,404 (91%) | 843 (89%) | 1,716 (89%) |
| controlled | 80 (8.8%) | 258 (9.1%) | 74 (7.2%) | 231 (8.8%) | 101 (11%) | 220 (11%) |
| Unknown | 26 | 83 | 22 | 85 | 27 | 64 |
| **ABC Control** | | | | | | |
| uncontrolled | 880 (95%) | 2,868 (99%) | 1,010 (96%) | 2,700 (100%) | 931 (96%) | 1,987 (99%) |
| controlled | 43 (4.7%) | 29 (1.0%) | 39 (3.7%) | 9 (0.3%) | 34 (3.5%) | 10 (0.5%) |
| Unknown | 8 | 12 | 6 | 11 | 6 | 3 |
| **ABCN Control** | | | | | | |
| uncontrolled | 898 (97%) | 2,879 (99%) | 1,023 (97%) | 2,707 (100%) | 936 (97%) | 1,992 (100%) |
| controlled | 26 (2.8%) | 21 (0.7%) | 28 (2.7%) | 6 (0.2%) | 30 (3.1%) | 6 (0.3%) |
| Unknown | 7 | 9 | 4 | 7 | 5 | 2 |

## Supplementary Table 4. Outcomes of Behavioural and Clinical Targets for Diabetes 2013-2023

| | *2013* | *2018* | *2023* | *Overall* |
|---|---|---|---|---|
| **BMI** | | | | |
| Mean (SD) | 24.6 (4.55) | 25.1 (4.61) | 25.2 (4.50) | 25.0 (4.56) |
| Median [Min, Max] | 24.2 [11.0, 72.9] | 24.7 [9.22, 79.6] | 24.8 [11.5, 66.0] | 24.6 [9.22, 79.6] |
| Missing | 397 (3.0%) | 561 (4.1%) | 799 (5.2%) | 1,757 (4.2%) |
| **height** | | | | |
| Mean (SD) | 156 (8.36) | 155 (8.37) | 156 (8.19) | 156 (8.31) |
| Median [Min, Max] | 155 [102, 199] | 154 [76.7, 187] | 155 [115, 187] | 155 [76.7, 199] |
| Missing | 378 (2.9%) | 520 (3.8%) | 793 (5.1%) | 1,691 (4.0%) |
| **weight** | | | | |
| Mean (SD) | 59.8 (12.2) | 60.4 (12.4) | 61.2 (12.3) | 60.5 (12.3) |
| Median [Min, Max] | 59.0 [19.0, 142] | 59.3 [25.0, 121] | 60.2 [18.1, 151] | 59.7 [18.1, 151] |
| Missing | 342 (2.6%) | 488 (3.6%) | 608 (3.9%) | 1,438 (3.4%) |
| **Treatment** | | | | |
| Not Treated | 4,039 (30.7%) | 1,500 (11.0%) | 1,253 (8.1%) | 6,792 (16.1%) |
| Treated Not Routine | 0 (0%) | 6,191 (45.3%) | 5,438 (35.3%) | 11,629 (27.5%) |
| Routine Treated | 9,119 (69.3%) | 5,968 (43.7%) | 8,716 (56.6%) | 23,803 (56.4%) |
| **Smoking Cessation** | | | | |
| Yes | 2,865 (21.8%) | 2,335 (17.1%) | 2,296 (14.9%) | 7,496 (17.8%) |
| No (active smoker) | 10,293 (78.2%) | 11,324 (82.9%) | 13,111 (85.1%) | 34,728 (82.2%) |
| **Fiber Intake** | | | | |
| Less than 3 Portion | 10,705 (81.4%) | 10,311 (75.5%) | 12,247 (79.5%) | 33,263 (78.8%) |
| 3 to 5 Portion | 2,138 (16.2%) | 2,734 (20.0%) | 2,693 (17.5%) | 7,565 (17.9%) |
| More than 5 Portion | 315 (2.4%) | 614 (4.5%) | 467 (3.0%) | 1,396 (3.3%) |
| **Physical Actibity** | | | | |
| <150 MET-min/week | 2,336 (17.8%) | 3,486 (25.5%) | 5,111 (33.2%) | 10,933 (25.9%) |
| 150–499 MET-min/week | 630 (4.8%) | 859 (6.3%) | 899 (5.8%) | 2,388 (5.7%) |
| ≥500 MET-min/week | 10,192 (77.5%) | 9,314 (68.2%) | 9,397 (61.0%) | 28,903 (68.5%) |
| **Blood Pressure Controle** | | | | |
| ≥140/≥90 mmHg | 3,909 (29.7%) | 4,264 (31.2%) | 4,369 (28.4%) | 12,542 (29.7%) |
| One <140 or <90 mmHg | 2,903 (22.1%) | 3,691 (27.0%) | 4,230 (27.5%) | 10,824 (25.6%) |
| <140/<90 mmHg | 6,346 (48.2%) | 5,704 (41.8%) | 6,808 (44.2%) | 18,858 (44.7%) |
| **Body Mass Index** | | | | |
| ≥27.5 | 2,883 (21.9%) | 3,454 (25.3%) | 3,897 (25.3%) | 10,234 (24.2%) |
| <18.5 or 23–27.4 | 5,926 (45.0%) | 5,986 (43.8%) | 6,756 (43.9%) | 18,668 (44.2%) |
| 18.5–22.9 (Normal) | 3,952 (30.0%) | 3,658 (26.8%) | 3,955 (25.7%) | 11,565 (27.4%) |
| Missing | 397 (3.0%) | 561 (4.1%) | 799 (5.2%) | 1,757 (4.2%) |
| **Waist Length** | | | | |
| Above risk cutoff | 3,630 (27.6%) | 4,298 (31.5%) | 5,662 (36.7%) | 13,590 (32.2%) |
| Intermediate risk | 3,600 (27.4%) | 4,117 (30.1%) | 4,488 (29.1%) | 12,205 (28.9%) |
| Below risk cutoff | 5,928 (45.1%) | 5,244 (38.4%) | 5,257 (34.1%) | 16,429 (38.9%) |
| **Composite Behavioral and Lab** | | | | |
| 0 | 17 (0.1%) | 82 (0.6%) | 70 (0.5%) | 169 (0.4%) |
| 1 | 366 (2.8%) | 906 (6.6%) | 920 (6.0%) | 2,192 (5.2%) |
| 2 | 1,927 (14.6%) | 3,156 (23.1%) | 3,316 (21.5%) | 8,399 (19.9%) |
| 3 | 4,047 (30.8%) | 4,299 (31.5%) | 4,906 (31.8%) | 13,252 (31.4%) |
| 4 | 3,751 (28.5%) | 2,954 (21.6%) | 3,520 (22.8%) | 10,225 (24.2%) |
| 5 | 2,037 (15.5%) | 1,345 (9.8%) | 1,478 (9.6%) | 4,860 (11.5%) |
| 6 | 601 (4.6%) | 344 (2.5%) | 385 (2.5%) | 1,330 (3.1%) |
| 7 | 15 (0.1%) | 12 (0.1%) | 13 (0.1%) | 40 (0.1%) |
| Missing | 397 (3.0%) | 561 (4.1%) | 799 (5.2%) | 1,757 (4.2%) |

## Supplementary Table 5. Outcomes of Laboratory Targets for Diabetes 2013-2023

| Outcome | 2013 | 2018 | 2023 |
|---|---|---|---|
| **FPG&OGTT Control Level** | | | |
| FPG ≥130 & OGTT ≥180 | 415 (44.6%) | 503 (47.7%) | 512 (52.7%) |
| Either FPG <130 or OGTT <180 | 196 (21.1%) | 232 (22.0%) | 228 (23.5%) |
| FPG <130 & OGTT <180 | 320 (34.4%) | 320 (30.3%) | 231 (23.8%) |
| **HBA1C Control Level** | | | |
| ≥9% | | | 359 (37.0%) |
| 7–8.9% | | | 151 (15.6%) |
| <7% | | | 225 (23.2%) |
| Missing | 931 (100%) | 1,055 (100%) | 236 (24.3%) |
| **Cholesterol Control Level** | | | |
| >250 mg/dL | 178 (19.1%) | 171 (16.2%) | 188 (19.4%) |
| 200–250 mg/dL | 335 (36.0%) | 343 (32.5%) | 441 (45.4%) |
| <200 mg/dL | 359 (38.6%) | 479 (45.4%) | 273 (28.1%) |
| Missing | 59 (6.3%) | 62 (5.9%) | 69 (7.1%) |
| **LDL Control Level** | | | |
| >130 mg/dL | 550 (59.1%) | 552 (52.3%) | 427 (44.0%) |
| 100–130 mg/dL | 214 (23.0%) | 298 (28.2%) | 273 (28.1%) |
| <100 mg/dL | 108 (11.6%) | 143 (13.6%) | 202 (20.8%) |
| Missing | 59 (6.3%) | 62 (5.9%) | 69 (7.1%) |
| **HDL Control Level** | | | |
| Male <30 / Female <40 | 110 (11.8%) | 153 (14.5%) | 239 (24.6%) |
| Male 30–39 / Female 40–49 | 272 (29.2%) | 343 (32.5%) | 352 (36.3%) |
| Male ≥40 / Female ≥50 | 490 (52.6%) | 497 (47.1%) | 311 (32.0%) |
| Missing | 59 (6.3%) | 62 (5.9%) | 69 (7.1%) |
| **Triglycerides Control Level** | | | |
| ≥200 mg/dL | 204 (21.9%) | 284 (26.9%) | 349 (35.9%) |
| 150–199 mg/dL | 164 (17.6%) | 181 (17.2%) | 221 (22.8%) |
| <150 mg/dL | 504 (54.1%) | 528 (50.0%) | 332 (34.2%) |
| Missing | 59 (6.3%) | 62 (5.9%) | 69 (7.1%) |
| **Chronic Kidney Disease Staging** | | | |
| Stage 5 (Kidney failure) | 2 (0.2%) | 3 (0.3%) | 5 (0.5%) |
| Stage 4 (Severe decrease) | 10 (1.1%) | 21 (2.0%) | 19 (2.0%) |
| Stage 3b (Moderate-severe) | 22 (2.4%) | 26 (2.5%) | 30 (3.1%) |
| Stage 3a (Mild-moderate) | 36 (3.9%) | 59 (5.6%) | 47 (4.8%) |
| Stage 2 (Mild decrease) | 203 (21.8%) | 231 (21.9%) | 181 (18.6%) |
| Stage 1 (Normal or high) | 599 (64.3%) | 653 (61.9%) | 620 (63.9%) |
| Missing | 59 (6.3%) | 62 (5.9%) | 69 (7.1%) |
| **lab_target_score** | | | |
| 0 | 48 (5.2%) | 67 (6.4%) | 87 (9.0%) |
| 1 | 148 (15.9%) | 189 (17.9%) | 214 (22.0%) |
| 2 | 205 (22.0%) | 231 (21.9%) | 271 (27.9%) |
| 3 | 211 (22.7%) | 218 (20.7%) | 182 (18.7%) |
| 4 | 153 (16.4%) | 171 (16.2%) | 103 (10.6%) |
| 5 | 82 (8.8%) | 89 (8.4%) | 33 (3.4%) |
| 6 | 25 (2.7%) | 28 (2.7%) | 12 (1.2%) |
| 7 | 0 (0%) | 0 (0%) | 0 (0%) |
| Missing | 59 (6.3%) | 62 (5.9%) | 69 (7.1%) |

## Supplementary Table 6. Weighted Average Score for Behavioural-Clinical Targets in Indonesia, 2013 – 2023

| Level | Name | 2013 | 2018 | 2023 | Change 2013-2023 | P-value |
|---|---|---|---|---|---|---|
| **Overall** | Overall | 3.56 (3.53–3.59) | 3.08 (3.05–3.11) | 3.14 (3.11–3.17) | -0.42 (-0.46–-0.37) | **<0.001** |
| **Island** | Java & Bali | 3.57 (3.53–3.61) | 3.08 (3.04–3.12) | 3.16 (3.11–3.20) | -0.41 (-0.47–-0.35) | **<0.001** |
| | Kalimantan | 3.55 (3.45–3.64) | 3.05 (2.96–3.14) | 3.09 (3.01–3.17) | -0.46 (-0.58–-0.34) | **<0.001** |
| | Lesser Sunda | 3.76 (3.63–3.89) | 3.29 (3.13–3.45) | 3.28 (3.10–3.45) | -0.49 (-0.70–-0.27) | **<0.001** |
| | Maluku & Papua | 3.37 (3.24–3.49) | 3.07 (2.92–3.21) | 2.97 (2.84–3.10) | -0.39 (-0.57–-0.21) | **<0.001** |
| | Sulawesi | 3.46 (3.38–3.54) | 3.18 (3.10–3.27) | 3.12 (3.04–3.19) | -0.35 (-0.46–-0.24) | **<0.001** |
| | Sumatera | 3.54 (3.48–3.60) | 3.01 (2.95–3.07) | 3.09 (3.03–3.14) | -0.45 (-0.53–-0.37) | **<0.001** |
| **Province** | Aceh | 3.63 (3.51–3.74) | 3.11 (3.00–3.23) | 3.16 (3.05–3.27) | -0.47 (-0.63–-0.31) | **<0.001** |
| | Bali | 3.80 (3.63–3.97) | 3.31 (3.14–3.48) | 3.56 (3.39–3.73) | -0.24 (-0.48–0.00) | 0.054 |
| | Banten | 3.58 (3.42–3.74) | 3.04 (2.87–3.22) | 3.12 (2.95–3.29) | -0.45 (-0.69–-0.22) | **<0.001** |
| | Bengkulu | 3.37 (3.15–3.60) | 3.12 (2.92–3.33) | 3.02 (2.81–3.22) | -0.36 (-0.66–-0.06) | **0.02** |
| | DKI Jakarta | 3.29 (3.10–3.48) | 2.95 (2.81–3.09) | 3.14 (2.98–3.29) | -0.15 (-0.40–0.09) | 0.215 |
| | Daerah Istimewa Yogyakarta | 3.59 (3.42–3.76) | 3.46 (3.29–3.63) | 3.44 (3.28–3.60) | -0.15 (-0.39–0.08) | 0.209 |
| | Gorontalo | 3.34 (3.15–3.54) | 3.27 (3.02–3.52) | 3.23 (3.03–3.43) | -0.11 (-0.40–0.17) | 0.425 |
| | Jambi | 3.43 (3.17–3.68) | 3.02 (2.79–3.25) | 3.14 (2.95–3.33) | -0.29 (-0.61–0.03) | 0.077 |
| | Jawa Barat | 3.48 (3.38–3.57) | 2.87 (2.77–2.97) | 3.09 (2.98–3.19) | -0.39 (-0.53–-0.25) | **<0.001** |
| | Jawa Tengah | 3.65 (3.57–3.73) | 3.19 (3.12–3.26) | 3.21 (3.13–3.28) | -0.45 (-0.56–-0.34) | **<0.001** |
| | Jawa Timur | 3.64 (3.57–3.71) | 3.18 (3.12–3.25) | 3.13 (3.06–3.20) | -0.51 (-0.61–-0.41) | **<0.001** |
| | Kalimantan Barat | 3.56 (3.39–3.73) | 3.24 (3.06–3.42) | 3.27 (3.11–3.42) | -0.29 (-0.52–-0.06) | **0.013** |
| | Kalimantan Selatan | 3.61 (3.46–3.75) | 3.17 (2.99–3.35) | 3.09 (2.95–3.23) | -0.51 (-0.71–-0.31) | **<0.001** |
| | Kalimantan Tengah | 3.52 (3.28–3.77) | 3.10 (2.89–3.30) | 2.84 (2.66–3.03) | -0.68 (-0.98–-0.37) | **<0.001** |
| | Kalimantan Timur | 3.56 (3.37–3.75) | 2.84 (2.69–3.00) | 3.04 (2.89–3.19) | -0.52 (-0.76–-0.27) | **<0.001** |
| | Kalimantan Utara | 3.19 (2.87–3.51) | 3.02 (2.70–3.34) | 3.18 (2.89–3.47) | -0.01 (-0.44–0.42) | 0.961 |
| | Kepulauan Bangka Belitung | 3.58 (3.36–3.81) | 3.26 (3.07–3.45) | 3.09 (2.94–3.24) | -0.50 (-0.76–-0.23) | **<0.001** |
| | Kepulauan Riau | 3.29 (2.91–3.67) | 2.99 (2.58–3.39) | 3.21 (3.02–3.41) | -0.08 (-0.50–0.35) | 0.716 |
| | Lampung | 3.94 (3.76–4.11) | 3.09 (2.93–3.26) | 3.03 (2.83–3.23) | -0.90 (-1.17–-0.64) | **<0.001** |
| | Maluku | 3.32 (3.10–3.54) | 3.06 (2.64–3.47) | 2.97 (2.74–3.20) | -0.35 (-0.67–-0.03) | 0.03 |
| | Maluku Utara | 3.48 (3.18–3.79) | 3.15 (2.87–3.44) | 3.30 (3.09–3.51) | -0.18 (-0.55–0.19) | 0.343 |
| | Nusa Tenggara Barat | 3.61 (3.39–3.83) | 3.25 (3.03–3.47) | 3.29 (3.04–3.55) | -0.32 (-0.65–0.02) | 0.069 |
| | Nusa Tenggara Timur | 3.89 (3.74–4.05) | 3.39 (3.21–3.58) | 3.25 (3.10–3.41) | -0.64 (-0.86–-0.43) | **<0.001** |
| | Papua | 2.93 (2.74–3.13) | 2.77 (2.43–3.11) | 2.96 (2.59–3.34) | 0.03 (-0.40–0.45) | 0.904 |
| | Papua Barat | 3.40 (2.82–3.99) | 3.39 (3.12–3.66) | 2.90 (2.46–3.35) | -0.50 (-1.24–0.24) | 0.186 |
| | Papua Barat Daya | 3.40 (3.06–3.74) | 3.11 (2.78–3.44) | 2.83 (2.46–3.20) | -0.57 (-1.07–-0.07) | **0.025** |
| | Papua Pegunungan | 3.88 (3.14–4.63) | 3.34 (2.66–4.02) | 3.19 (2.70–3.68) | -0.69 (-1.58–0.20) | 0.126 |
| | Papua Selatan | 3.04 (2.57–3.52) | 2.84 (2.43–3.25) | 2.74 (2.33–3.15) | -0.31 (-0.93–0.32) | 0.335 |
| | Papua Tengah | 4.13 (3.78–4.48) | 3.08 (2.58–3.59) | 2.82 (2.41–3.23) | -1.31 (-1.85–-0.77) | **<0.001** |
| | Riau | 3.47 (3.29–3.65) | 3.00 (2.84–3.16) | 3.12 (2.99–3.26) | -0.35 (-0.57–-0.13) | **0.002** |
| | Sulawesi Barat | 3.52 (3.26–3.78) | 3.39 (3.06–3.73) | 2.91 (2.70–3.13) | -0.60 (-0.94–-0.27) | **<0.001** |
| | Sulawesi Selatan | 3.42 (3.28–3.55) | 3.22 (3.07–3.36) | 3.15 (3.01–3.29) | -0.27 (-0.46–-0.07) | **0.007** |
| | Sulawesi Tengah | 3.60 (3.41–3.79) | 3.13 (2.96–3.29) | 3.24 (3.09–3.40) | -0.36 (-0.60–-0.11) | **0.004** |
| | Sulawesi Tenggara | 3.63 (3.40–3.86) | 3.35 (3.10–3.59) | 3.17 (3.01–3.34) | -0.46 (-0.74–-0.18) | **0.001** |
| | Sulawesi Utara | 3.43 (3.30–3.57) | 3.03 (2.88–3.17) | 2.90 (2.77–3.03) | -0.53 (-0.72–-0.34) | **<0.001** |
| | Sumatera Barat | 3.41 (3.25–3.57) | 3.09 (2.93–3.24) | 3.38 (3.24–3.52) | -0.03 (-0.25–0.18) | 0.774 |
| | Sumatera Selatan | 3.49 (3.32–3.66) | 2.84 (2.61–3.07) | 3.35 (3.21–3.48) | -0.14 (-0.36–0.07) | 0.191 |
| | Sumatera Utara | 3.51 (3.39–3.62) | 2.94 (2.80–3.08) | 2.83 (2.71–2.94) | -0.68 (-0.84–-0.52) | **<0.001** |

## Supplementary Table 7. Weighted Average Score for Laboratory Targets in Indonesia, 2013 - 2023

| Name | 2013 | 2018 | 2023 | Change 2013-2023 | P-value |
|---|---|---|---|---|---|
| **Overall** | **2.72 (2.58–2.86)** | **2.81 (2.71–2.92)** | **2.42 (2.29–2.55)** | **-0.30 (-0.50–-0.11)** | **0.002** |
| Java & Bali | 2.68 (2.49–2.88) | 2.82 (2.71–2.93) | 2.46 (2.29–2.63) | -0.23 (-0.49–0.03) | 0.087 |
| Kalimantan | 2.33 (1.89–2.77) | 2.61 (2.01–3.22) | 2.32 (2.03–2.61) | -0.01 (-0.53–0.52) | 0.977 |
| Lesser Sunda | 3.81 (3.33–4.30) | 2.87 (2.09–3.64) | 2.37 (1.84–2.91) | -1.44 (-2.17–-0.72) | <0.001 |
| Maluku & Papua | 3.27 (2.81–3.73) | 4.37 (3.64–5.10) | 3.13 (2.49–3.77) | -0.14 (-0.92–0.65) | 0.728 |
| Sulawesi | 2.49 (2.10–2.88) | 2.70 (2.28–3.12) | 2.31 (2.03–2.58) | -0.18 (-0.66–0.29) | 0.455 |
| Sumatera | 2.93 (2.63–3.22) | 2.69 (2.24–3.14) | 2.28 (2.05–2.51) | -0.65 (-1.02–-0.27) | <0.001 |

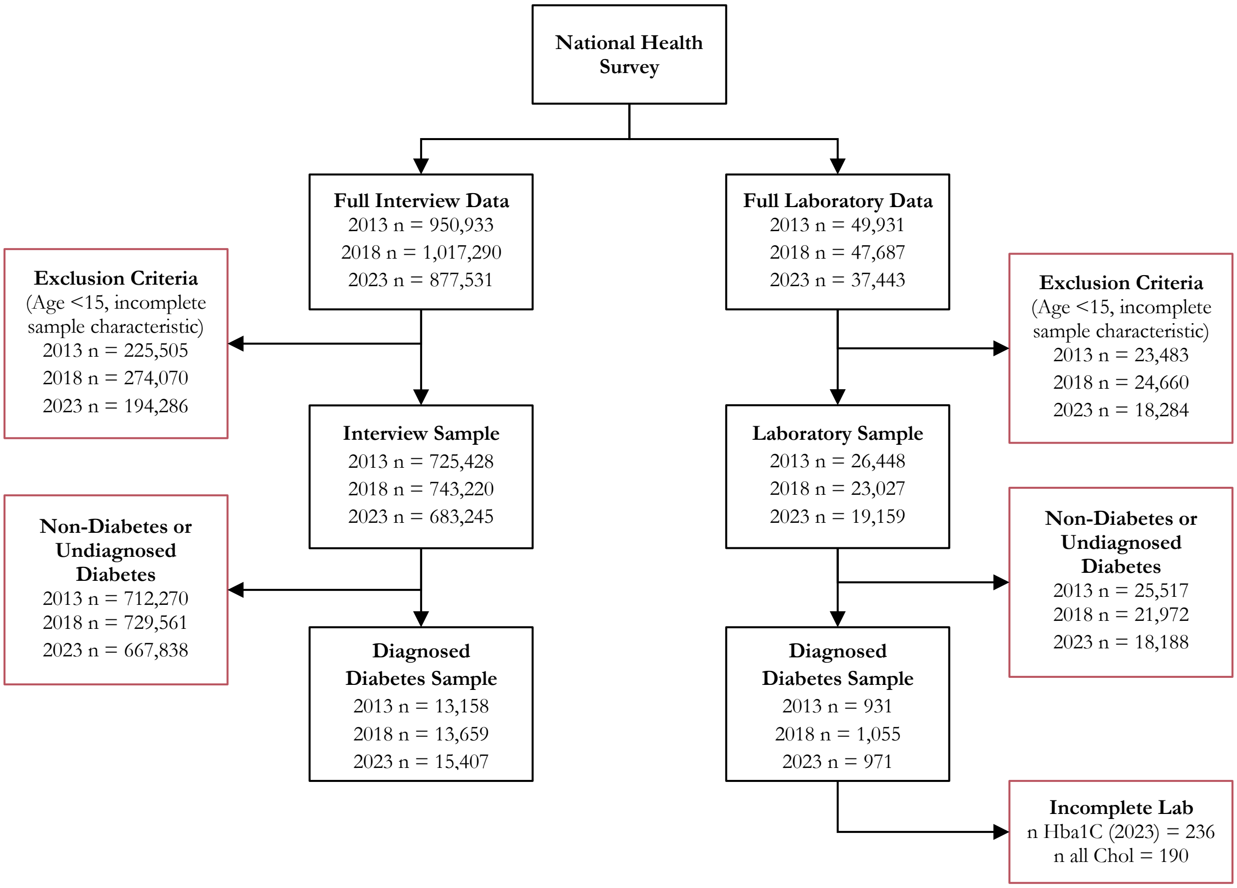


**Supplementary Figure 1 Flowchart of Interview and Laboratory Sample Selection and Exclusion for Diabetes Analysis, RISKESDAS 2013 and 2018, and SKI 2023**
Flowchart of participant selection for diabetes analysis in RISKESDAS 2013 and 2018, and SKI 2023, showing interview respondents, those with laboratory data, and exclusions due to missing glucose measures or incomplete covariates (wealth status, education).

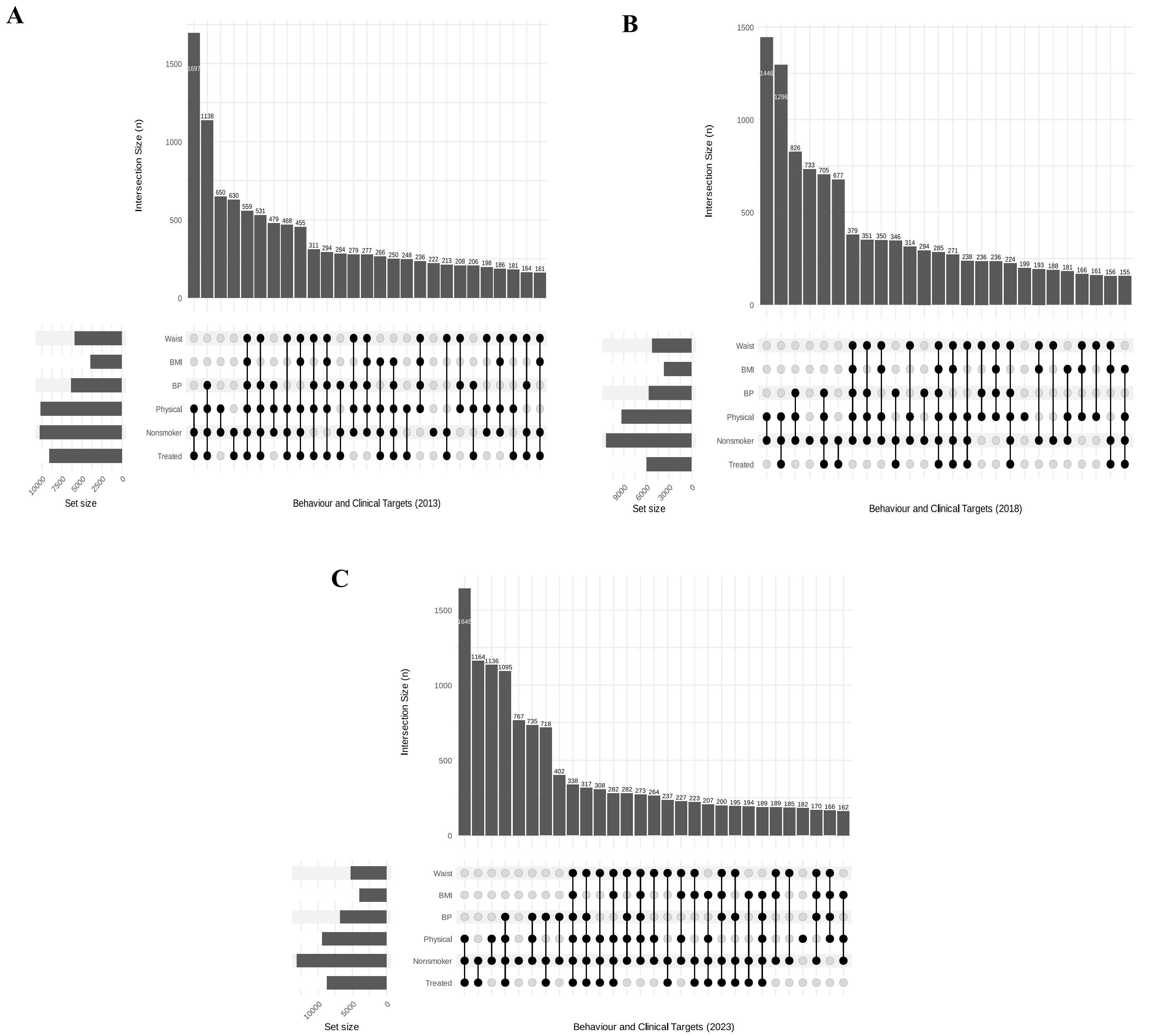


**Supplementary Figure 2 Patterns of Simultaneous Achievement in Behavioral and Clinical Diabetes Care Targets, Indonesia (2013–2023)** UpSet plots showing the most common combinations of key diabetes care indicators across three survey years **A**) 2013,**B**) 2018, and **C**) 2023. Each bar in the top histogram represents the number of individuals achieving a specific combination of targets. The connected dots below each bar indicate which indicators are included in the combination. Indicators assessed include waist circumference control (Waist), body mass index control (BMI), sufficient physical activity (Physical), normal fasting glucose (NormoGlu), and current treatment for diabetes (Treated). Horizontal bars on the left side of each plot show the total number of individuals achieving each individual target, regardless of other target achievements.

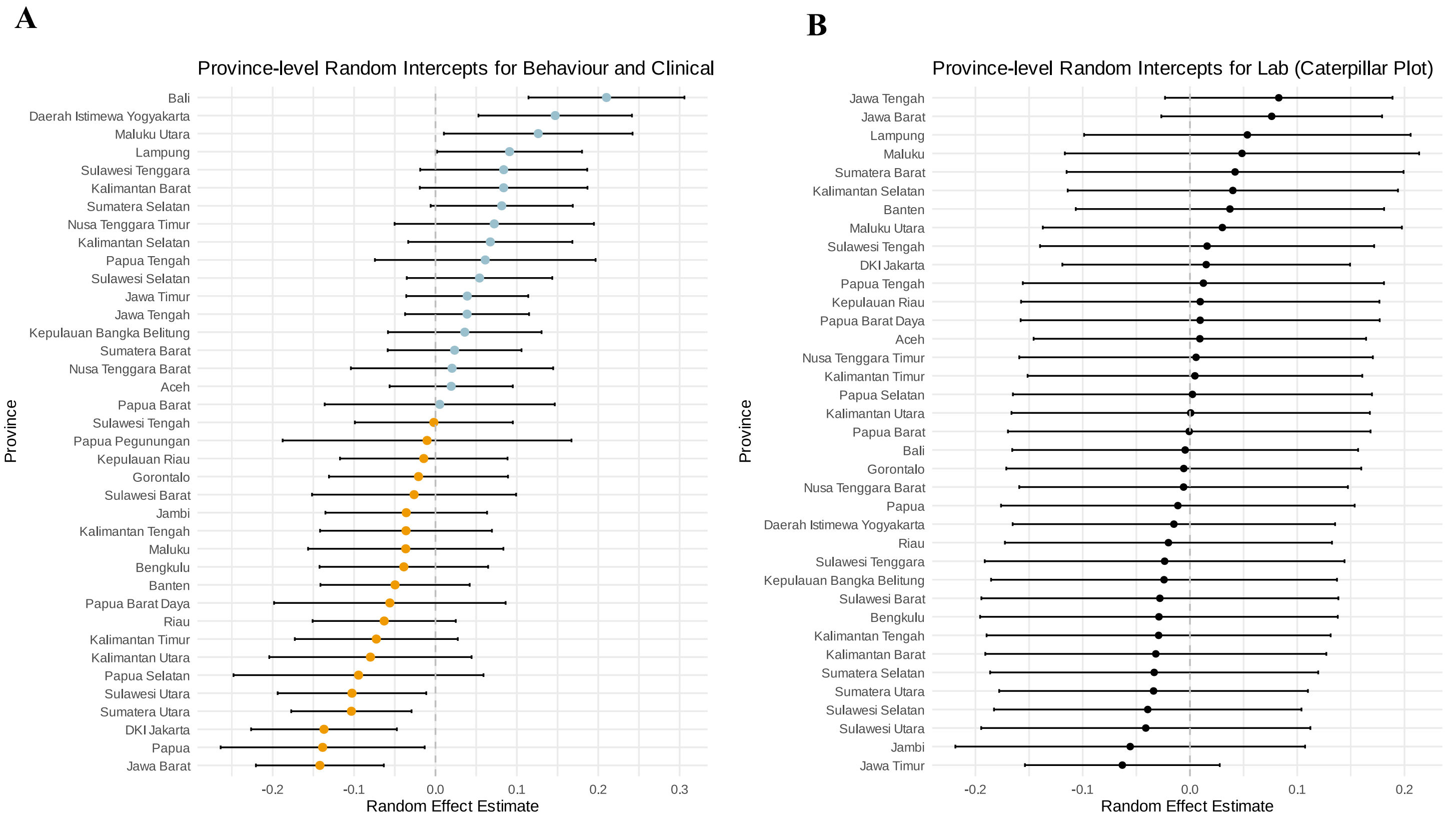


**Supplementary Figure 3 Province-Level Variation in Diabetes Care Performance: Random Intercepts from Multilevel Models.** Caterpillar plots display the estimated random intercepts (and 95% confidence intervals) from multilevel models of diabetes care outcomes across Indonesian provinces. **A)** Behavioural and clinical care steps. **B)** Lab-based outcome Estimates above zero indicate better-than-average provincial performance after adjusting for individual-level covariates.

# Harmonization of Survey Questionnaire on Behavioral Target

| **2013** | | | **2018** | | | **2023** | | |
|---|---|---|---|---|---|---|---|---|
| **Data code** | **Question** | **Answer Labels** | **Data code** | **Question** | **Answer Labels** | **Data code** | **Question** | **Answer Labels** |
| **B12** | Have [NAME] ever been diagnosed with diabetes Mellitus/ diabetes by a doctor? | Yes | **B10B06** | Have [NAME] ever been diagnosed with diabetes Mellitus/ diabetes by a doctor? | 1. Yes | **B07A** | Have [NAME] ever been diagnosed with diabetes Mellitus/ diabetes by a doctor? | Yes |
| | | Not | | | 2. No | | | Not |
| | | | **B10B07** | What age was first diagnosed with diabetes mellitus/ diabetes? | | **B08** | B08. What age was [NAME] first diagnosed diabetes mellitus (DM)/ diabetes? | |
| **B13C** | Taking Anti-Diabetic Drugs | Yes | **B10B08** | What type of treatment does [NAMA] obtain? | 1. Anti-DM (OAD) Drugs from medical personnel | **B09A** | B09. What type of treatment does [NAMA] obtain? | Yes |
| | | Not | | | 2. Insulin injection | | a. Anti-DM (OAD) Drugs from medical personnel | Not |
| **B13D** | Insulin injection | Yes | | | 3. Anti-DM (OAD) drugs from medical personnel and insulin injections | **B09B** | B09. What type of treatment does [NAMA] obtain? | Yes |
| | | Not | | | 4. Untreated | | b. Insulin injection | Not |
| | | | | | | **B09C** | B09. What type of treatment does [NAMA] obtain? | Yes |
| | | | | | | | c. Self-purchased Anti-DM (OAD) drugs | Not |
| | | | | | | **B09D** | B09. What type of treatment does [NAMA] obtain? | Yes |
| | | | | | | | d. Not yet need Anti-DM Drugs (OAD) | Not |

| 2013 | | | 2018 | | | 2023 | | |
|---|---|---|---|---|---|---|---|---|
| **Data code** | **Question** | **Answer Labels** | **Data code** | **Question** | **Answer Labels** | **Data code** | **Question** | **Answer Labels** |
| | | | **B10B09** | Do [NAME] take/inject anti-diabetic medications Routinely as directed by your doctor? | 1. Yes, according to doctor's instructions | **B10** | B10. Do [NAME] take/inject anti-diabetic medications according to the doctor's instructions? | Yes, according to doctor's instruction s |
| | | | | | 2. Inappropriate | | | Not according to doctor's instruction s |
| | | | **B10B10A** | Why didn't [NAMA] take the medicine as directed by the doctor? | 1. Yes | **B11** | B11. MAIN Reason [NAME] not to take/inject DM medication | |
| | | | | a. Often forgetting | 2. No | | according to the doctor's instructions? | |
| | | | **B10B10B** | b. Medicines are not available in health facilities | 1. Yes | | Feeling healthy | |
| | | | | (Hospital/Puskesmas/Phar macy) | 2. No | | Medicines are not available at health facilities (Hospitals/Puskesmas/Ph armacies) | |
| | | | **B10B10C** | c. Taking traditional medicine | 1. Yes | | Not resistant to the side effects of the drug | |
| | | | | | 2. No | | Taking traditional medicine | |
| | | | **B10B10D** | d. Not resistant to the side effects of the drug | 1. Yes | | Bored/lazy/often forgotten | |
| | | | | | 2. No | | DM medication is only taken during pregnancy/postpartum | |
| | | | **B10B10E** | e. Unable to afford medication regularly | 1. Yes | | Other | |
| | | | | | 2. No | | | |
| | | | **B10B10F** | f. Not routinely going to a service facility for treatment | 1. Yes | | | |

| 2013 | | | 2018 | | | 2023 | | |
|---|---|---|---|---|---|---|---|---|
| **Data code** | **Question** | **Answer Labels** | **Data code** | **Question** | **Answer Labels** | **Data code** | **Question** | **Answer Labels** |
| | | | | health | 2. No | | | |
| | | | **B10B10G** | g. Feeling healthy | 1. Yes | | | |
| | | | | | 2. No | | | |
| | | | **B10B10H** | h. Others, mention | 1. Yes | | | |
| | | | | ...................................... | 2. No | | | |
| | | | **B10B10HS** | Mentioned.........................<br>............. | | **B11S** | B11. Mentioned | |
| | | | | | | **B12** | B12. Does [NAME] recheck (control) | Yes, routine |
| | | | | | | | DM disease to health care facilities? | Yes, sometimes |
| | | | | | | | | Not |
| **B13A** | Does [NAME] currently do the following This is to control diabetes | | **B10B11A** | What does [NAME] do to control diabetes mellitus? | 1. Yes | **B14A** | B14. What [NAME] does to control | Yes |
| | Diet | Yes | | a. Dining arrangements | 2. No | | Diabetes mellitus? a. Dining arrangements | Not |
| | | Not | | | | | | |
| **B13B** | Sports | Yes | **B10B11B** | What does [NAME] do to control diabetes mellitus? | 1. Yes | **B14B** | B14. What [NAME] does to control | Yes |
| | | Not | | b. Sports | 2. No | | Diabetes mellitus? b. Sports | Not |
| | | | **B10B11C** | What does [NAME] do to control diabetes mellitus? | 1. Yes | **B14C** | B14. What [NAME] does to control | Yes |
| | | | | c. Herbal alternatives | 2. No | | Diabetes mellitus? c. Herbal alternatives | Not |

| 2013 | | | 2018 | | | 2023 | | |
|---|---|---|---|---|---|---|---|---|
| **Data code** | **Question** | **Answer Labels** | **Data code** | **Question** | **Answer Labels** | **Data code** | **Question** | **Answer Labels** |
| | | | B10G17 | G17 Has [NAME] ever smoked? | Yes, every day Yes, not every dayNever smoke | G11 | G11. Did [NAME] ever smoke? | Yes, every day yes, not every dayNever smoke |
| G06 | How old [NAME] is to start smoking every day | Range: 1-98 | B10G18 | G18 What age did [NAME] start smoking every day? | Range: 7-40 | G12 | G12. How old is [NAME] to start smoking every day? | Don't Know/Forget |
| G07 | How old [NAME] was when you first smoked | Range: 1-98 | B10G19 | G19 How old was [NAME] when I first smoked? | Range: 7-40 | G13 | G13. How old was [NAME] when you first smoked? | Don't Know/Forget |
| G23 | G23 Usually in 1 week, how many days [NAME] eat fresh fruits? (USE PROPS) | Range: 0-7 | B10G08 | Usually in 1 week, how many days [NAME] eats fresh fruits? | Range: 0-7 | G25 | G25. Usually in 1 week, how many days [NAME] eat fresh fruits? | Range: 0-7 |
| G24 | G24 What is the average serving [NAME] of fresh fruits consumed in one day of those days ? (USE CART | Range: 1-5 | B10G09 | What is the average serving [NAME] consuming fresh fruits in one day of those days ? | Range: 0.5-7.0 | G26 | G26. What is the average serving [NAME] consuming fresh fruits in one day of those days ? | Range: 0.1-7.0 |
| G16 | G16 Is [NAME] used to do strenuous physical activity, which is done continuously for at least 10 minutes s | 12 | B10G29 | G29. Is [NAMA] used to do strenuous physical activity, which is done continuously for at least 10 minutes each time? | YES | G36 | G36. Is [NAMA] used to do strenuous physical activity, which is done continuously for at least 10 minutes each time? | YesNo |

| 2013 | | | 2018 | | | 2023 | | |
|---|---|---|---|---|---|---|---|---|
| **Data code** | **Question** | **Answer Labels** | **Data code** | **Question** | **Answer Labels** | **Data code** | **Question** | **Answer Labels** |
| G17 | G17 How many days of the week do you usually do that strenuous physical activity? | Range: 1-7 | B10G30 | G30. Usually how many days a week, [NAME] do that strenuous physical activity? | Range: 1-7 | G37 | G37. Usually how many days a week, [NAME] do that strenuous physical activity? | Range: 1-7 |
| G18_JAM | G18 Usually in a day, how long does [NAME] do such strenuous physical activity? | Range: 0-9 | B10G31J | G31. Usually in a day, how long does [NAME] do such strenuous physical activity? [HOURS] | Range: 0-10 | G38A | G38. Usually in a day, how long does [NAME] do such strenuous physical activity? a.Hours | Range: 0-16 |
| G18_MNT | Minute | Range: 0-40 | B10G31M | G31. Usually in a day, how long does [NAME] do such strenuous physical activity? [MINUTES] | Range: 0-45 | G38B | G38.b.Minutes | Range: 0-48 |
| G19 | G19 Is [NAME] used to moderate physical activity , which is done continuously for at least 10 minutes | 12 | B10G32 | G32 Is [NAMA] used to do moderate physical activity , which is done continuously for at least 10 minutes each time? | YesNo | G39 | G39. Is [NAMA] used to do moderate physical activity , which is done continuously for at least 10 minutes each time? | 12 |
| G20 | G20 How many days of the week do you usually do moderate physical activity? | Range: 1-7 | B10G33 | G33 How many days of the week do you usually do moderate physical activity? | Range: 1-7 | G40 | G40. Usually how many days a week, [NAME] do moderate physical activity? | Range: 1-7 |
| G21_JAM | G21 Usually in a day, how long does [NAME] do moderate physical activity? | Range: 0-9 | B10G34J | G34 Usually in a day, how long does [NAME] do the moderate physical activity? | Range: 0-13 | G41A | G41. Usually in a day, how long does [NAME] do the moderate physical activity? a.Hours | Range: 0-21 |

| 2013 | | | 2018 | | | 2023 | | |
|---|---|---|---|---|---|---|---|---|
| **Data code** | **Question** | **Answer Labels** | **Data code** | **Question** | **Answer Labels** | **Data code** | **Question** | **Answer Labels** |
| G21_MNT | Minute | Range: 0-45 | B10G34M | G34 Usually in a day, how long does [NAME] do the moderate physical activity? | Range: 0-59 | G41B | G41.b.Minutes | Range: 0-59 |
